# Based on the Results of PEDV Phylogenetic Analysis of the Most Recent Isolates in China, the Occurrence of Further Mutations in the Antigenic Site S1° and COE of the S Protein Which Is the Target Protein of the Vaccine

**DOI:** 10.1155/2023/1227110

**Published:** 2023-02-22

**Authors:** Xin Yao, Yu Zhu, Wen-Ting Qiao, Wei-Hong Lu, Yu-Qian Zhang, Jin-Long Li

**Affiliations:** ^1^College of Veterinary Medicine, Northeast Agricultural University, Harbin 150030, China; ^2^Key Laboratory of the Provincial Education Department of Heilongjiang for Common Animal Disease Prevention and Treatment, Northeast Agricultural University, Harbin 150030, China; ^3^Heilongjiang Key Laboratory for Laboratory Animals and Comparative Medicine, Northeast Agricultural University, Harbin 150030, China

## Abstract

The continuous challenge of existing vaccine systems by porcine epidemic diarrhea virus (PEDV) variant strains in recent years, it caused significant economic losses in the global swine industry. A PEDV virulent strain CH/HLJBQL/2022 was successfully isolated in China in this study. A genome-wide based phylogenetic analysis suggests that CH/HLJBQL/2022 belongs to the GII subtype, and 96.3%–99.6% homology existed in the whole genomes of other strains. For the first time, simultaneous mutations of four amino acids were found in the highly conserved membrane (M) and nucleocapsid (N) proteins, as well as five amino acid mutations that differed from the vast majority of strains in the spike (S) protein. Mutations in the M and S proteins were found to produce coils with different angles by building 2D and 3D structural models. Epitope analysis indicated that the isolates produced specific changes and that the transmembrane function of the M protein had not been affected. In addition, typing markers exist during strain evolution, but isolates are using the fusion of specific amino acids from multiple variant strains to add additional features, as also demonstrated by protein alignments and 3D models of numerous subtype strains. These results suggest that aa mutations in the M and S proteins may have changed the structure and antigenic epitope of the isolates and PEDV is evolving again on the basis of variants that have been found to counteract the immune network of the new vaccine.

## 1. Introduction

Porcine epidemic diarrhea virus (PEDV) is an enveloped virus with a 28 kb single-stranded positive-sense RNA and belongs to the *α* Coronavirus spp [1]. The genome in turn consists of 5′UTR-replicase polyprotein 1a/b (ORF1a/b) -spike (S)-ORF3-envelope (E)-membrane (M)-nucleocapsid (N)-3′UTR. PEDV, the leading cause of piglet diarrhea in the world, was first reported in Europe in 1977, became widespread in East Asia after 2011, and in the Americas after 2013, causing great economic losses to the swine industry [2, 3].

As the virus continues to evolve, scholars refer to the strains that are close to CV777 as classical (GI) and those that are distant (GII) [4]. But for a more detailed division, some scholars believe that named sequentially according to the evolutionary tree, there are divided into GIIa and GIIb, there are reports that GI and GII are divided into three groups a, b, and *c* each, and also the variants divided into Asian mutant strains geographically, American virulent strains and American indels strains, different [1, 5, 6].

Protein S is a type of important protein. It is a type I glycoprotein composed of subunits S1 and S2 of the trimer on the surface of the virus. It mediates the entry of PEDV into cells by binding to the expected receptor, aminopeptidase N, and sialic acid [7, 8]. S1 is involved in receptor binding and S2 in the viral envelope and target cell membrane fusion [9]. The reason for the immune escape of the virus from the host after vaccination is that mutations, deletions, and insertions in the S protein can alter the epitope for that antigen. [10]. The M protein, the most abundant membrane glycoprotein of the viral capsule, which is mostly located inside the capsule membrane, and only a small region of the amino terminus that is glycosylated exposed outside the capsule membrane, is an important protein for viral particle assembly and budding, which possesses a candidate antigen for PEDV genetically engineered vaccine because of its ability to induce interferon production [11]. The N protein is linked to virion RNA and plays an important role in the synthetic genomic process of the virus, it can bind to the membrane and promote the assembly and replication of new virions and is very critical for the induction of cellular immunity [12].

In the present study, novel mutations were generated in the S protein and the highly conserved N and M proteins of the isolates compared with the strains of each subtype, and mutations in the S and M proteins produced coils in the three-dimensional structure and resulted in antigenic epitope changes. Identical amino acid mutations were found in different genotypes, implying that PEDV evolution was purposeful and constrained, whereas this study found that newly emerging isolates had newly added mutations on the basis of typing markers, which were collectively patched by very few strains in other temporal spaces, providing hypotheses for the emergence of new genotypes. Taken together, these results provide a further complement to the detection and evolution of PEDV and will facilitate further research on the prevention and treatment of PEDV.

## 2. Materials and Methods

### 2.1. Specimen Collection and Pathogen Identification

In 2021, small intestinal tissues were collected from a diarrhoeal pig farm in Heilongjiang Province, China, with clinical symptoms of watery diarrhea, vomiting, dehydration, and rapid weight loss. The intestinal contents of infected piglets from the same house were mixed, and sample nucleic acids were extracted using Trizol™ (Thermo, USA) following the manufacturer's instructions. The gDNA Eraser-treated RNA samples were reverse-transcribed with strand-specific RT primers at 42°C for 15 min with the PrimeScript® Reverse Transcriptase (Takara, China). The strand-specific quantitative PCR (qPCR) was performed with gene-specific primers and the LightCycler® 480 SYBR Green I Master (Roche, Switzerland) on the QuantStudio™ 5 Real-Time PCR Detection System (Thermo, USA). ORF3 plasmid, flat used as an internal control to normalize gene expression was kept by the laboratory.

### 2.2. Cell Lines and Virus Isolation

Cells were maintained in Dulbecco's modified Eagle's medium (DMEM, Hyclone, USA) supplemented with 10% fetal bovine serum (Hyclone, USA). The small intestines and their contents, which tested positive by RT-PCR, were homogenized and made into 20% suspensions using DMEM and 100 U/mL penicillin-streptomycin (Hyclone, USA) at 4°C for 3000 × *g* for ten minutes, 8000 × *g* for one minute after aspirating the supernatant. The supernatant was collected and passed through a 0.22 *μ*m filter (Millipore, Billerica, MA, USA) and stored as virus adsorbate at −80°C freezer after filtration. When Vero E6 cells (Harbin Veterinary Research Institute, Chinese Academy of Agricultural Sciences, China) were grown to 80% confluence in T25 (Corning, USA) flasks, they were rinsed twice using PBS, inoculated with 2 mL of adsorbate, and supplemented with 5 *μ*g/mL pancreatin (Hyclone, USA). After incubation at 37°C for 2 h, the adsorbent solution was discarded and the cells were rinsed twice using PBS and incubated in 5 mL DMEM supplemented with 2% serum and 5 *μ*g/mL pancreatin at 37°C in 5% CO_2_ for 72 h to 84 h. Cultures were placed at −80°C for repeated freeze-thawing three times, and the mixture was mixed using a 0.22 *μ*m filter after marking P1 passage, the blind passage was performed after a positive RT-qPCR test, and cytopathological effects were observed after the tenth passage.

### 2.3. Construction of ORF3 Plasmids

ORF3 primers were designed based on the sequence of CV777 (AF353511.1) published at the National Center for Biotechnology Information (NCBI), the RNA of PEDV CV777 (Harbin Pharmaceutical Group Holding, China) was subjected to PCR using ORF3-F/R, the PCR products were recovered (TIANGEN, China) and ligated to T-Vector pMD19 (Simple, Takara, China) according to the manufacturer's instructions, and the plasmids from cultured single colonies were extracted and Sanger sequenced (Tsingke Biotechnology, China), and the sequencing results were consistent with the database (Supplementary Table 1).

### 2.4. One-Step Growth Curve Was Plotted

And the one-step growth curve of PEDV was determined with viral titers expressed as 50% tissue culture infectious dose (TCID_50_). Vero E6 cells (2 × 10^6^/mL) were seeded into 6-well cell culture plates and incubated in a 5% CO_2_ incubator for 24 h. Vero E6 cells were then inoculated with PEDV at a multiplicity of infection (MOI) of 1.0 for time point cultures separately up to 72 h. Co-culture for 24 h was selected as the experimental group, and cells cultured in DMEM were used as the control group.

### 2.5. Indirect Immunofluorescence Assay (IFA)

Vero E6 cells in six-well plates (Corning, China) at 80% confluence were infected with PEDV CV777 and CH/HLJBQL/2022 (OM914738.1) for 24 h and then fixed using 4% paraformaldehyde for 30 min. After washing the cells three times using PBS, cells were perforated with 0.2% TritonX-100 (Beyotime, China) for 10 min, and after washing three times, blocking was performed by incubation with 0.3% Bovine Serum Albumin Fraction V (BSA, Sigma, USA) at 37°C for 30 min. Washed three times with PBS and incubated with mouse anti-PEDV N protein monoclonal antibody (Medgene Labs, SD-2–5, USA) for 1 h at 37°C. After three washes, Alexa Fluor 488 (Beyotime, China) conjugated goat anti-mouse IgG was added, incubated for 30 min at 37°C in dark conditions, washed three times, and cells were viewed using an inverted fluorescence microscope (Leica, Germany).

### 2.6. Genomic Sequencing of PEDV CH/HLJBQL/2022

The acquisition of a viral second strand was consistent with the method of pathogen identification used before library preparation using Nextera XT reagents (Illumina) and sequencing on the NovaSeq 6000 (Illumina, USA) at the Shanghai Tanpu Biotechnology Co., Ltd. (Tpbio, China). To remove sequencing adaptors and low-quality reads, raw data were filtered and trimmed by Fastp (v0.20.0). The alignment of the obtained sequencing data was performed with BBmap (v38.51) to the NCBI NT database to remove corresponding rRNA, host, and bacterial sequences. De novo genome assembly was performed using SPAdes (v3.14.1) and SOAPdenovo (v2.04). These extracted assembled reads limited the minimum contig length to 100 bases with the best BLAST hits to the NCBI NT database.

### 2.7. Sequence Analysis

Multiple protein amino acid sequences of the reference strain (Supplementary Table 2) and CH/HLJBQL/2022 were aligned using the DNAMAN (v6.0) software. The neighbor-joining (NJ) method of MEGA (v6.0) software was used to establish phylogenetic trees for the whole genome and each protein, and the bootstrap value was set to 1000 replicates. ITOL participated in the process of phylogenetic tree change of strains. Genomic and individual gene nucleotide homologies for the reference strain and CH/HLJBQL/2022 were analyzed using the MegAlign program in DNASTAR (v7.1.0.44), and the results were analyzed via OmicShare for Heatmap production.

### 2.8. Protein 3D Structure Model and Function Prediction

Homology modeling of the respective protein tertiary structures was performed using Phyre2 (https://www.sbg.bio.ic.ac.uk/phyre2/html) and SWISS-MODEL (https://swissmodel.expasy.org). At the same time, Phyre2 validates the above DNAMAN alignment results for the amino acid sequence of each protein. FirstGlance in JMOL (https://proteopedia.org/wiki/fgij/index.htm) verified the amino acid mutation position, and SWISS-MODEL verified the effect of the mutation on the structure. TMHMM (v2.0, https://services.healthtech.dtu.dk) was used to predict the transmembrane functional changes of the S protein and M protein.

### 2.9. Statistical Analysis

Statistical comparisons were performed using GraphPad Prism (version 8.3) software. Student's *t*-test was used to analyze the data. A *P* value < 0.05 was considered statistically significant. The error bars represent the standard error (±SE). Fluorescence imaging quantitative analysis was performed using ImageJ (version 1.8). Differential coefficients less than 0.5 were considered statistically significant in the heatmap.

## 3. Results

### 3.1. Replication and Cell Adaptation of CH/HLJBQL/2022

**Figure 1:**
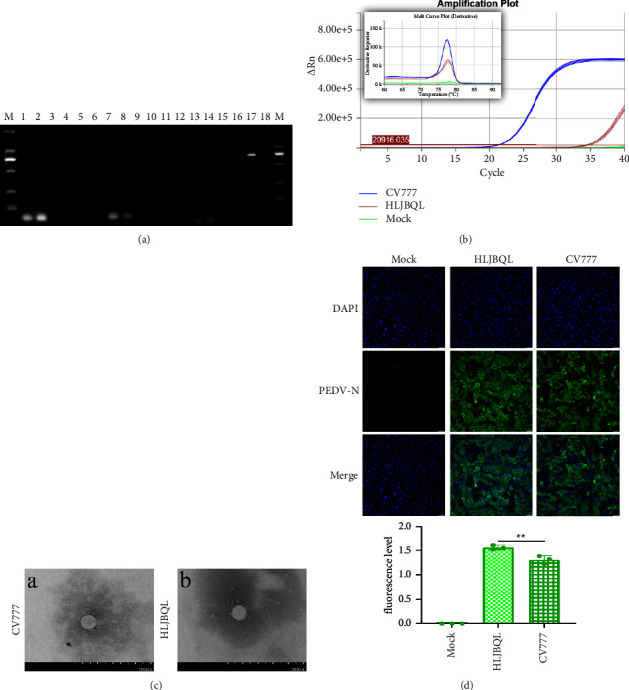
Isolation and identification of the PEDV CH/HLJBQL/2022 strain. (a) Assays for PCV2, PDCoV, TGEV, PRRSV, PBoV, PRV, PKV, BVDV, and PEDV were performed sequentially, with the complex number being the singular negative control, using 2000 marker as the standard. (b) Small intestines and their contents, which tested positive for PEDV, were made up as an adsorbent solution, and Vero E6 cells were infected, and it was observed that after shedding of the cells freeze-thawed three times and the mixture was tested. Domesticated CV777 served as a positive control. (c) A. Transmission electron microscopy of Vero E6 cell supernatant infected with CV777. B Transmission electron microscopy of Vero E6 cell supernatant infected with CH/HLJBQL/2022. (d) Immunofluorescence results of mock infected group, isolate infected group, and CV777 infected group. Infected cells were detected using a monoclonal antibody against PEDV N protein and Alexa Fluor 488 conjugated goat anti-mouse IgG and quantified using ImageJ (*n* = 3, *P* < 0.005).

**Figure 2:**
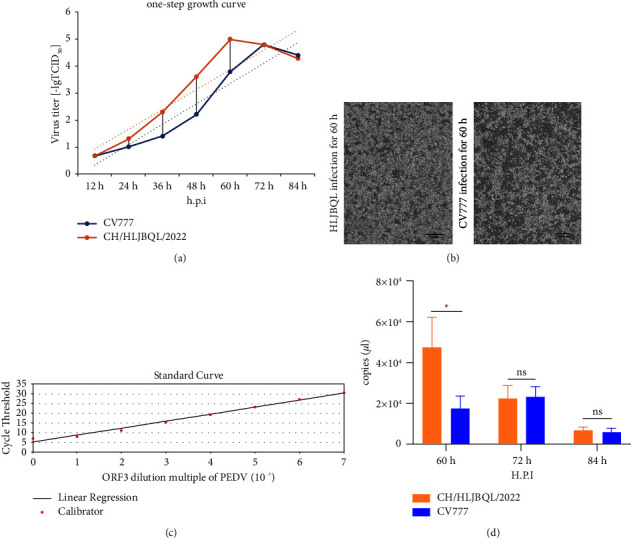
Virulence testing of the PEDV CH/HLJBQL/2022 strain. (a) The TCID_50_ was determined every 12 h from 12 h to 84 h and the values of the 7 assays were combined to plot the one-step growth curve of the virus, with CV777 as the positive control (MOI = 1). (b) CH/HLJBQL/2022 and CV777-infected Vero E6 cells passaged to 10 passages were observed microscopically for 60 h. (c) ORF3 recombinant plasmids were prepared to develop a standard curve by tenfold dilution detection. (d) Viral load assays were performed at 60 h, 72 h, and 84 h of CH/HLJBQL/2022 infection, and CV777 served as a positive control (*n* = 3, *P* < 0.05).

To determine diarrhea antigens collected at the Heilongjiang pig farm, China, total RNA was extracted from the small intestine and its contents, and the RT-PCR results indicated that the samples were positive for PEDV (Figure 1(a) and Supplementary Table 3). The samples were homogenized and made into an adsorbent fluid to infect Vero E6 cells, RT-qPCR was performed on the first passage venom, and the results showed that the isolates were successfully replicated, and the domesticated CV777 was used as a positive control (Figure 1(b)). Using transmission electron microscopy to visualize the viral fluid when the virus was cultured to the tenth passage, it could be clearly observed that a 100 nm sized PEDV virion with a crown-like fibrous process on the vesicle membrane could be observed (Figure 1(c)). After the isolates were infected with Vero E6 cells for 24 h, IFA showed specific fluorescence using a monoclonal antibody against the PEDV N protein, which was absent in the negative control (Figure 1(d)). Quantification results showed that the isolates had adapted, using CV777 as a positive control. Based on the above results, we named CH/HLJBQL/2022. The TCID_50_ of it was determined by the Reed̶Muench method and a one-step growth curve was plotted, showing that this strain was significantly more efficient in replication in vitro than the classical strain (Figure 2(a)). Microscopic observation at 60 h of infection verified this phenomenon (Figure 2(b)). To better explain this phenomenon, we constructed ORF3 recombinant plasmids targeting conserved sequences of PEDV and plotted a standard curve (Figure 2(c)). Viral load measurements were performed on Vero E6 cells infected for 60 h, 72 h, and 84 h, respectively, and the results were compatible with the growth curves (Figure 2(d)). CH/HLJBQL/2022 was more virulent than the classical strains before 72 h, and after 72 h, the virions were gradually inactivated, lysed in a 37°C incubator, and probably due to the death of the host.

### 3.2. Complete Genome Sequence of CH/HLJBQL/2022

**Figure 3:**
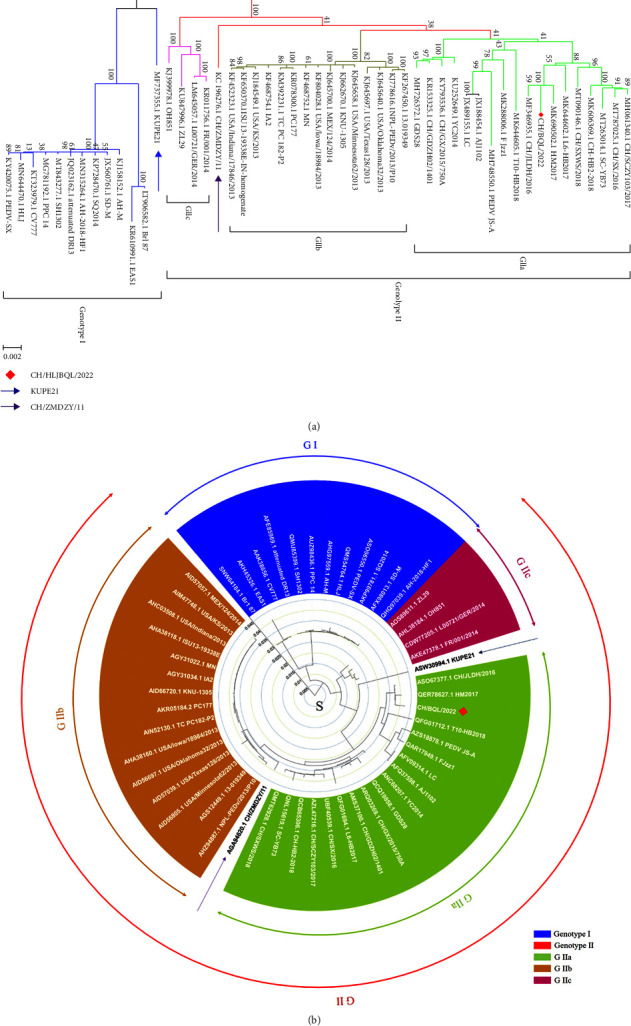
The whole genome and S protein sequences of the CH/HLJBQL/2022 strain were analyzed for genetic evolution. (a) The CH/HLJBQL/2022 and 50 PEDV strains were subjected to evolutionary analysis and divided into two subtypes, GI and GII, with GII further subdivided into GIIa, GIIb, and GIIc. CH/HLJBQL/2022 is marked in red, and arrows indicate KUPE21 (MF737355.1) and CH/ZMDZY/11 (KC196276.1) as early fusion strains. (b) The evolutionary tree analysis of the S proteins of the 51 viruses was performed, and the results were generally consistent with the genome-wide evolutionary analysis.

**Figure 4:**
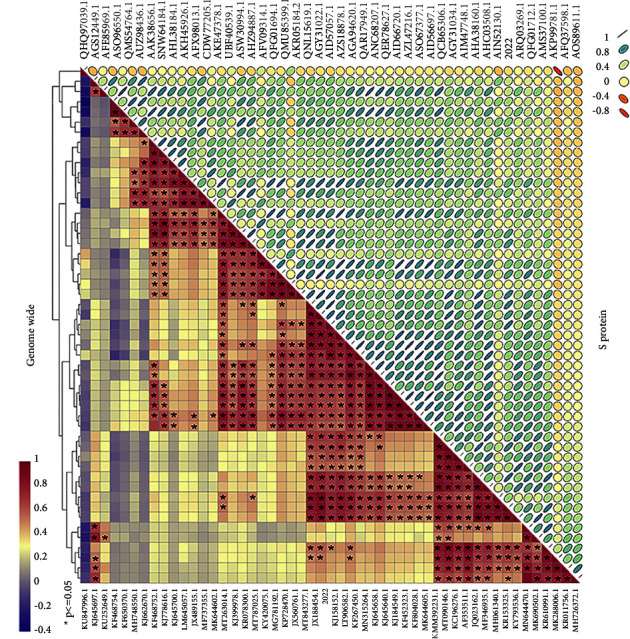
Analysis of the entire genome and homology of the S protein sequence of strain CH/HLJBQL/2022. (a) The whole genomes of 51 viruses were analyzed for homology, and the homology results were subjected to a normalized heatmap. (b) The S protein was processed as above. Differential coefficients greater than 0.5 were considered statistically significant in the heatmap.

The complete genome sequence of CH/HLJBQL/2022 was deduced using the Illumina platform and submitted to GenBank with the login number OM914738.1 (Supplementary Figure 1). A total of 28095 nucleotides were detected for this strain, including ORF1a (nt 276–12629), ORF1b (nt 12659–20620), S (nt 20617–24774), ORF3 (nt 24774–25448), E (nt 25429–25659), M (nt 25667–26347), and N (nt 26359–27684). The full gene phylogenetic analysis showed that CH/HLJBQL/2022 belongs to the GIIa subtype (Figure 3(a) and Supplementary Table 2), which is consistent with the developmental analysis of the S gene (Figure 3(b)). It has also been maintained at some distance from classical strains in evolutionary analyses of ORF3, E, N, and M proteins, although the M and N proteins are relatively conserved and there are no differences in small protein E (Supplementary Figure 2). Genome-wide homology analysis indicated that CH/HLJBQL/2022 shared 96.3%–99.5% identity with other strains and was most similar to HM2017, while CV777 was shown to share 96.7% nucleotide homology (Supplementary Figures 2 and 3). The homology normalized heat map shows significant differences when compared to the genomes of other strains, and this result was repeated for S and other proteins (Figure 4 and Supplementary Figure 4).

### 3.3. Amino Acid Mutations of CH/HLJBQL/2022

**Figure 5:**
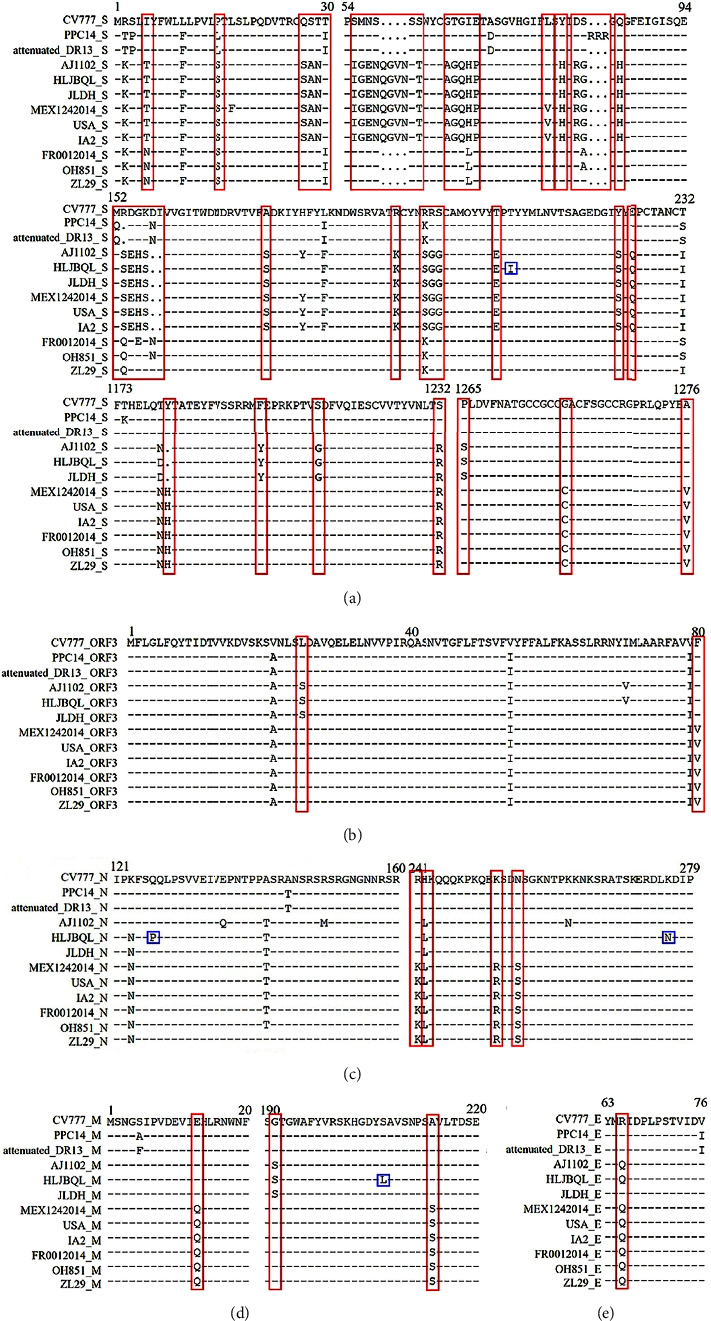
CH/HLJBQL/2022 was aligned with the amino acid sequences of other types of representative strains. Three viruses of each of the GI subtype, GIIb subtype, and GIIc subtype, two viruses of the GIIa subtype, and a total of nine viruses from CH/HLJBQL/2022 were randomly selected for alignment. (a) S protein to contrast. (b) ORF3 protein to contrast. (c) N protein to contrast. (d) M protein to contrast. (e) E protein to contrast. The red box represents the characteristics of each subtype, and the blue box represents the unique evolutionary characteristics of CH/HLJBQL/2022.

To determine the specificity of CH/HLJBQL/2022, amino acid alignments, and analyses were performed using DNAMAN on three randomly selected GI, GIIb, and GIIc strains and two additional GIIa strains. The results showed that five amino acid mutations, T-I (aa 213), K-N (aa 568), D-Y (aa 571), and AS-VI (aa 888̶889), were generated located on the S protein (Figure 5(a) and Supplementary Figure 5). Three and one amino acid mutations, Q-P (aa 126), K-N (aa 276), I-T (aa 402), and S-L (aa 207), resulted from being located in the N and M proteins, respectively (Figures 5(b)–5(e) and Supplementary Figure 6).

The results also showed that there was a certain amount of amino acid-specific conservation, including S, N, M, and ORF3 proteins, among the grouped strains, and these phenomena were the basis for the identification of PEDV grouped strains. Examples are GI/GIIc group TRCY in the S protein, and GIIa/b group TKCY (aa 190–193). GI/GIIb/c group SLD in the ORF3 protein, GIIa group SSD (aa 24–26). GI group RH in the N protein, GIIa group RL, GIIb/c group KL (aa 241-242). GI/GIIa group IEH in the M protein, and GIIb/c group IQH (aa 12–14) (Figure 5). The segregation of CH/HLJBQL/2022 implies that PEDV is attempting novel mutations to include proteins traditionally thought to be highly conserved while reducing the primer accuracy for previous typing assays.

### 3.4. Mutations of 4-aa Altered S and M Protein Spatial Structure

**Figure 6:**
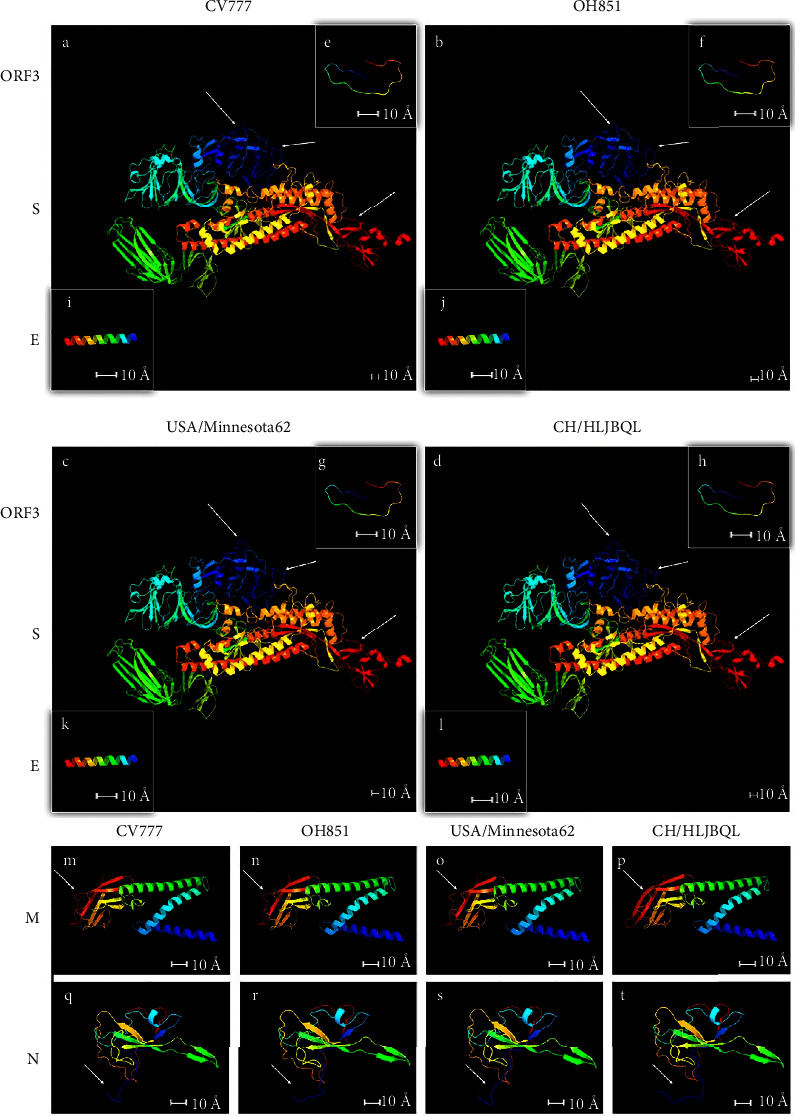
Protein tertiary structure modeling was performed on four representative strains of GI, GIIa, GIIb, and GIIc. (a–d) S protein 3D homology simulation modeling. Two differences were found between GI/GIIc and GIIa/b, and one difference was between GI/GIIb/GIIc and GIIa. (e–h) ORF3 protein 3D homology simulation modeling. (i–l) E protein 3D homology simulation modeling. (m–p) M protein 3D homology simulation modeling. At the end of the M protein, GIIa is longer than the other isoforms *α* Helix. (q–t) N protein 3D homology simulation modeling. The differential bending of the prosegment of the N protein, as revealed by inspection, is due to a homology modeling algorithm and has no practical implications.

One strain from each subtype was selected as a representative for protein spatial structure modeling, which revealed the presence of three helical structural alterations in the S protein through four sets of subtype contrasts in GI/GIIc and GIIa/b, and in GI/GIIb/c and GIIa, where the M protein *α* helix is elongated and distinguished from other genotypes (Figures 6(a)–6(d) and 6(m)–6(p)). No significant changes were found for ORF3, E, and N proteins (Figures 6(e)–6(l) and 6(q)–6(t)). Highly conserved amino acids within the genotypes produced divergent coils and elongated *α* helix in the M and S proteins, consistent with our typing landmark results derived from the sequence alignments described above (Supplementary Figures 8A, 8C, and 8E).

To further determine the alterations brought about by the unique amino acid mutations, structural prediction of CH/HLJBQL/2022 for nine amino acids that differed from the other strains was performed using DNAMAN, along with a detailed alignment of the tertiary structures (Supplementary Figure 7). The results showed that M protein S-L (aa 207) and S protein T-I (aa 213) and AS-VI (aa 888-889) four amino acids caused coil changes in M protein and N protein, respectively, which were verified by the establishment of the tertiary structure by SWISS-MODEL and JMOL (Supplementary Figures 8B, 8D, and 8F).

### 3.5. The 5-aa Alters the S Antigen Epitope

**Figure 7:**
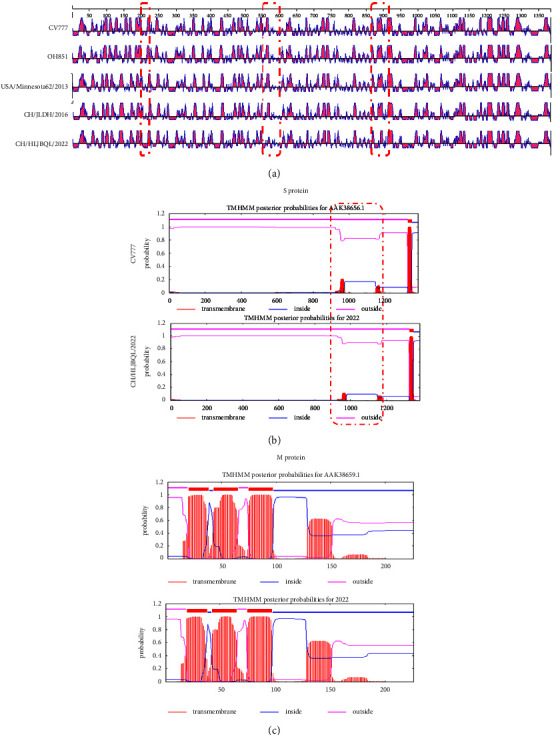
S protein antigenic epitopes S and M protein transmembrane function prediction. (a) Antigenic epitope analysis was performed on four subtype representative strains and CH strains with three red boxes denoting 5-aa mutations of S protein, T-I (aa 213), K-N (aa 568), D-Y (aa 571), and AS-VI (aa 888-889). (b) S protein transmembrane function prediction. The red box indicates the level of transmembrane capacity of the S2 subunit, with CV777 as a control. (c) Prediction of M protein transmembrane function.

The CH/HLJBQL/2022 specific antigenic epitope was found to be distinguished from other strains using DNASTAR, after S protein sequence specificity and structural specificity. Aa 213, aa 568–571, and aa 888-889 showed significant differences from the currently identified PEDV, with T-I (aa 213) and AS-VI (aa 888-889) fitting the sequence and resulting results (Figure 7(a)). Although the mutation of S protein AS-VI (aa 888-889) alters protein transmembrane prediction compared to CV777, there are no actual functional changes (Figure 7(b)). The same is true for the transmembrane function prediction of the M protein (Figure 7(c)).

## 4. Discussion

PEDV has become one of the leading causes of viral diarrhea and causes great losses to the swine industry worldwide. A genome-wide CH/S of PEDV was reported in China in 1986, and since 2011, PEDV has continued to be endemic in China [13]. Initiated in 2013, continues to be first detected and prevalent in the United States. As of December 2021, the whole genomes of 811 for all isolates of PEDV have been reported at NCBI, with the highest number being 299 isolates from China, followed by 232 isolates from the United States. At the end of the 20th century, PEDV CV777 vaccines were developed, and these inactivated or attenuated vaccines were already widely used in regional pig farms in China and made important contributions to the early control of PEDV infection in China. However, newly reported PEDV variant strains account for the vast majority since the Chinese and U.S. pandemics in 2011 and 2013, and vaccines derived from classical strains cannot provide sufficient protection against currently circulating strains due to viral mutations [14, 15]. Therefore, field monitoring and analysis of PEDV genes will help to understand the trends of PEDV and help to develop more effective control measures.

The S gene is a commonly used molecular marker in the study of genomic characteristics of PEDV strains. It consists of the S1 antigenic region and the S2 membrane fusion region and contains four neutralizing epitopes COE (aa 499–638), SS2 (aa 748–755), SS6 (aa 764–771), and 2c10 (aa 1368–1374) [16]. In addition to the four recognized neutralizing epitopes, there are many discovered epitopes such as S1° (aa 1–219), E10E-1-10 (aa 435–485), S1B (aa 510–640), P4B-1 (aa 575–639), and S1D (aa 636–789), among others [16–18]. Mutations in the S protein may alter its antigenic, pathogenic, and neutralizing properties [19]. The detection of amino acid changes in the PEDV s protein; therefore, helps to understand the evolutionary characteristics of PEDV. In the present study, it was discovered for the first time that the S protein generated 5-aa mutations in the S1°, COE, and aa 888 –889 regions simultaneously, resulting in CH/HLJBQL/2022 in the S1° and aa 888 –889 regions of (Supplementary Figures 8B, 8D, and 8F). At the same time, we speculate that the antigenic epitope of CH/HLJBQL/2022 appears as a change that distinguishes all subtypes, in two regions of neutralizing epitopes that have been found and one that has not. This could have a negative impact on a large proportion of the currently available PEDV vaccines (a weak strain vaccine generated against CV777 and a pandemic strain vaccine generated against AJ1102). In recent years, with the continuous emergence of mutant strains, researchers have confirmed that existing commercial vaccines (GI) are unable to provide adequate immune protection against the currently circulating strains (G2), and our findings not only confirm this view but also reveal that the strains are constantly evolving on a G2 basis, which is a great challenge for our existing G1-G2 junction system.

Due to the immune pressure of vaccines and to preserve the immune evasion capacity, the S protein of the virus undergoes frequent alterations [20]. The M protein plays an important role in the assembly and budding of viral particles and has been a candidate antigen for vaccines due to its ability to mediate interferon production by the body [21]. The latest studies have shown that seven neutralizing epitopes are newly identified on the M protein, which implies the possibility of making a vaccine, but unfortunately, the M protein, which has always been considered relatively conserved, has been found by us in the CH/HLJBQL/2022 due to alterations in the amino acid sequence and spatial structure [22]. The technical accuracy of the initial use of the N protein to establish a molecular biological diagnosis has been challenged, as has been the case with the M protein (Figure 5).

Recombination has been shown to be an important means of virus evolution, and genetic recombination is a common phenomenon among coronaviruses [23]. KUPE21 (MF737355.1) and CH/ZMDZY/11 (KC196276.1) were the products of preliminary recombination. Two viruses provided recombinant genomes, but the reassortant virus was not of the maternal subtype. Among the first nine simultaneous amino acid mutations in which we found CH/HLJBQL/2022, the AS-VI (aa 888 – 889) mutation findings in the S protein were consistent with the CH/GZZY/12/2020 (MZ161063.1) strain collected in the Shaanxi Province in 2020. The S-L (aa 207) mutation findings in the M protein were consistent with the JS-2013 (MH910099.1) strain collected in Shanghai in 2013 and the GDS43 (MH726381.1) strain collected in Guangdong Province in 2015. The mutation in the N protein K-N (aa 276) was found to be consistent with the 17LS0201 (MF118919.1) strain collected in Zhejiang Province in 2017, and the I-T (aa 402) mutation was found for the first time. CH/HLJBQL/2022 is caused by 4 viruses across space and time involving 3 proteins with little likelihood of recombination, so we speculate that the flag sequence is present within the protein and highly conserved across subtypes [24], and now PEDV is attempting to break through this logical relationship. These results suggest that the original means of detection and vaccine efficacy are being challenged, PEDV infection is becoming more and more complicated in China, and that future studies on the genetic evolution of new circulating PEDV strains in China are warranted.

In this study, PEDV strains were identified from Chinese pig farms in November 2021 and classified into the GIIa subgroup. Compared with classical strains, CH/HLJBQL/2022 is unique in gene and has many variations in neutralizing epitopes, which indicates that the development of new vaccines based on these new PEDV variants may be a necessary condition to control the prevalence of PEDVs in China. In addition, in this study, changes in relatively conservative proteins are a major challenge to the original detection methods and candidate vaccine development. All the facts indicate that PEDV is breaking through the original cognition and moving towards a more complex path. Our results provide valuable information for the prevention and treatment of PEDV and will help to further the study of the evolutionary law.

## Data Availability

The strain data used to support the findings of this study are included within the supplementary information files. The protein 3D model data used to support the findings of this study are available from the corresponding author upon request.

## Supplementary Materials

Supplementary Materials
*Supplementary Table 1.* Recombinant plasmid sequences of PEDV ORF3. *Supplementary Table 2.* PEDV strains were used in this study. *Supplementary Table 3.* The primer sequences. *Supplementary Figure 1* Sequencing results of CH/HLJBQL/2022. (A) Contig-depth statistical results are presented. (B) Best alignment results display. (C) The assembly result circle diagram exhibits. CDs: CDs fragment after assembled sequence annotation; GC content: the display of GC content variation across assembled sequences (sliding windows of varying lengths were selected based on sequence length; contig length < 10000, sliding window length < 50; contig length < 100000, sliding window length 500); GC skew±: GC content offset, GC skew = (G − C)/(G + C), which measures the relative content of G and C, gives a positive value for GC skew if G > C and a negative value for G. *Supplementary Figure 2.* Evolutionary analysis of 51 PEDV strains. (A) Evolutionary analysis of the ORF3 protein. CH/HLJBQL/2022 is marked in red, and arrows indicate KUPE21 (MF737355.1) and CH/ZMDZY/11 (KC196276.1) as early fusion strains. (B) Evolutionary analysis of the N protein. (C) Evolutionary analysis of E protein. (D) Evolutionary analysis of M protein. *Supplementary Figure 3*. Sequence homology analysis of the whole genome of strain CH/HLJBQL/2022. *Supplementary Figure 4*. The homology of ORF3, E, M, and N sequences of strain CH/HLJBQL/2022 was analyzed and displayed by heat map normalization. (A) Results of the ORF3 gene sequence homology thermogram. (B) Results of the E gene sequence homology thermogram. (C) Results of the M gene sequence homology thermogram. (D) Results of the N gene sequence homology thermogram. *Supplementary Figure 5*. 11 representative strains and CH/HLJBQL/2022 strain S protein sequence alignment. CV777 (AF353511.1), PPC 14 (MG781192.1), attenuated DR13 (JQ023162.1), FR/001/2014 (KR011756.1), OH851 (KJ399978.1), ZL29 (KU847996.1), IA2 (KF468754.1), MEX/124/2014 (KJ645700.1), USA/Minnesota62/2013 (KJ645658.1), AJ1102 (JX188454.1) and CH/JLDH/2016 (MF346935.1) were aligned with reference. *Supplementary Figure 6*. 11 representative strains and CH/HLJBQL/2022 strain other proteins sequence alignment. (A) Gene nucleotide contrast for ORF3. (B) Gene nucleotide contrast for E. (C) Gene nucleotide contrast for M. (D) Gene nucleotide contrast for N. *Supplementary Figure 7*. The secondary structures of S, N, and M proteins were predicted. (A) The amino acid mutation sites were four predicted as strands and one as a coil in the S protein. (B) The amino acid mutation sites were predicted to be helical in two helixes and coils in the N protein in one. (C) The site of the amino acid mutation was predicted to be coil in the M protein. *Supplementary Figure 8*. Tertiary structure modeling of mutation sites. (A) The ASSS-ASSG signature distinguishing GIIa from GI/GIIb/C is found in the M protein. This mutation, located at aa 191-226, produced a peculiar coil. (B) An S-L mutation (aa 207) in the M protein, found in the CH/HLJBQL/2022 strain, produced a peculiar coil. (C) The GGC-RSC, SYQ-YYE, and TKCY-TRCY motifs GIIa/b and GI/GIIc distinctions are found in the S protein. These mutations located at aa 160-241 produced a peculiar coil with an extended last hallmark *α* Helix. (D) The S-L mutation (aa 213) found in the CH/HLJBQL/2022 strain that neutralizes an epitope in S1° of the S protein gives rise to a peculiar coil. (E) The distinction between SVI̶SFI and GVS̶GAS hallmarks GI and GII is found in the S protein. These mutations, located at aa 861̶961, produced special coils and *α* Helix. (F) The VI-AS mutation (aa 888 –889) in the S protein, found in the CH/HLJBQL/2022 strain, produced a special coil.
