# Supplementary material for: Based on the Results of PEDV Phylogenetic Analysis of the Most Recent Isolates in China, the Occurrence of Further Mutations in the Antigenic Site S1° and COE of the S Protein Which Is the Target Protein of the Vaccine
Source: Transbound Emerg Dis. 2023 Feb 22;2023:1227110. doi: 10.1155/2023/1227110 (PMC12016877; doi:10.1155/2023/1227110)
Supplement: Supplementary Materials — Supplementary Table 1. Recombinant plasmid sequences of PEDV ORF3. Supplementary Table 2. PEDV strains were used in this study. Supplementary Table 3. The primer sequences. Supplementary Figure 1 Sequencing results of CH/HLJBQL/2022. (A) Contig-depth statistical results are presented. (B) Best alignment results display. (C) The assembly result circle diagram exhibits. CDs: CDs fragment after assembled sequence annotation; GC content: the display of GC content variation across assembled sequences (sliding windows of varying lengths were selected based on sequence length; contig length < 10000, sliding window length < 50; contig length < 100000, sliding window length 500); GC skew±: GC content offset, GC skew = (G − C)/(G + C), which measures the relative content of G and C, gives a positive value for GC skew if G > C and a negative value for G. Supplementary Figure 2. Evolutionary analysis of 51 PEDV strains. (A) Evolutionary analysis of the ORF3 protein. CH/HLJBQL/2022 is marked in red, and arrows indicate KUPE21 (MF737355.1) and CH/ZMDZY/11 (KC196276.1) as early fusion strains. (B) Evolutionary analysis of the N protein. (C) Evolutionary analysis of E protein. (D) Evolutionary analysis of M protein. Supplementary Figure 3. Sequence homology analysis of the whole genome of strain CH/HLJBQL/2022. Supplementary Figure 4. The homology of ORF3, E, M, and N sequences of strain CH/HLJBQL/2022 was analyzed and displayed by heat map normalization. (A) Results of the ORF3 gene sequence homology thermogram. (B) Results of the E gene sequence homology thermogram. (C) Results of the M gene sequence homology thermogram. (D) Results of the N gene sequence homology thermogram. Supplementary Figure 5. 11 representative strains and CH/HLJBQL/2022 strain S protein sequence alignment. CV777 (AF353511.1), PPC 14 (MG781192.1), attenuated DR13 (JQ023162.1), FR/001/2014 (KR011756.1), OH851 (KJ399978.1), ZL29 (KU847996.1), IA2 (KF468754.1), MEX/124/2014 (KJ645700.1), USA/Minnesota62/2013 ( [file 1227110.f1.zip › Supplementary Material-1227110 (2).docx]

***Supplementary Material***

**Based on the results of PEDV phylogenetic analysis of the most recent isolates in China, the occurrence of further mutations in the antigenic site S1 and COE of the S protein which is the target protein of the vaccine**

**Xin Yao^a^, Yu Zhu^a^, Wen-Ting Qiao^a^, Wei-Hong Lu^a^, Yu-Qian Zhang^a^, Jin-Long Li ^a,b,c,^***

^a^ College of Veterinary Medicine, Northeast Agricultural University, Harbin, 150030, PR China

^b^ Key Laboratory of the Provincial Education Department of Heilongjiang for Common Animal Disease Prevention and Treatment, Northeast Agricultural University, Harbin, 150030, PR China

^c^ Heilongjiang Key Laboratory for Laboratory Animals and Comparative Medicine, Northeast Agricultural University, Harbin, 150030, PR China

* Corresponding author

**Jin-Long Li**

College of Veterinary Medicine, Key Laboratory of the Provincial Education Department of Heilongjiang for Common Animal Disease Prevention and Treatment, Heilongjiang Key Laboratory for Laboratory Animals and Comparative Medicine, Northeast Agricultural University, Harbin, 150030, PR China.

E-mail address: Jinlongli@neau.edu.cn (J.-L. Li)

**Supplementary Table 1.** Recombinant plasmid sequences of PEDV ORF3.

| pMD19-ORF3: |
| --- |
| TCATTCACTAATTGTAGCATACTCGTCTAGTTGAATTGAGTCAAATGCAGCATTAGTAATGCCAACAATTTGATGTTGCGAAAAGACATAAAGCTTCTTGCCATCAAGAAGCTCAACAGTTCGCAACAGCTGTAGGTCGGCTTCTTGCCGCCCACGTATAGCTAGATACAAGTCAATGCTACTAACAAAAGCAACAAAAGAGTTGCCAAAAGTGATGTAATGGTCACCACCTTCTAAAATCACAATGGATTTGCCGTCATAATAAGCTGCTTTACCATTGAGGAAAGAAAGTGTCGTAGTATTAAAAATAATAAAGAGCGCATTTTTATAGCGCCAGGAGTAAAAGCAGACTAAACAAAGCCTGCCAATAAGTGTGCAACAAATAATAGTTGCATCTAAAAATGCACCACAATAATATAAAAGTGGGCAATAAAGAACAATGACAGCAAAACGCGCTGCCAACATAATATAATTGCGCCTCAAAGAAGACGCTTTAAACAGTGCAAAGAAGTAGATAAAAACACTGGTGAAAAGAAAACCCGTCACATTTGAAGCTTGTCTAATTGGAACTACATAGAGCTCCAACTCTTGGACAGCATCCAAAGACAAGTTAGCAGACTTTGAGACATCTTTGACAACTGTGTCAATCGTGTATTGAAAAAGTCCAAGAAACAT |

**Supplementary Table 2.** PEDV strains used in this study.

| Accession | Isolate | Collection Date | Geo Location | Genotype |
| --- | --- | --- | --- | --- |
| MF737355.1 | KUPE21 | 2001 | South Korea | - |
| KC196276.1 | CH/ZMDZY/11 | 2011 | China | - |
| AF353511.1 | CV777 | 1977 | Belgium | GI |
| LT906582.1 | Br1/87 | 1987 | United Kingdom | GI |
| JQ023162.1 | attenuated DR13 | 2009 | South Korea | GI |
| KJ158152.1 | AH-M | 2011 | China | GI |
| JX560761.1 | SD-M | 2012 | China | GI |
| MT843277.1 | SH1302 | 2013 | China | GI |
| KR610991.1 | EAS1 | 2014 | Thailand | GI |
| MG781192.1 | PPC 14 | 2014 | South Korea | GI |
| KP728470.1 | SQ2014 | 2014 | China | GI |
| MN644470.1 | HLJ | 2015 | China | GI |
| KY420075.1 | SX | 2015 | China | GI |
| MN315264.1 | AH-2018-HF1 | 2018 | China | GI |
| JX188454.1 | AJ1102 | 2011 | China | GIIa |
| MK288006.1 | FJzz1 | 2011 | China | GIIa |
| JX489155.1 | LC | 2011 | China | GIIa |
| MH726372.1 | GDS28 | 2012 | China | GIIa |
| MH748550.1 | JS-A | 2012 | China | GIIa |
| KR153325.1 | CH/GDZH02/1401 | 2014 | China | GIIa |
| KU252649.1 | YC2014 | 2014 | China | GIIa |
| KY793536.1 | CH/GX/2015/750A | 2015 | China | GIIa |
| MF346935.1 | CH/JLDH/2016 | 2016 | China | GIIa |
| MK690502.1 | HM2017 | 2016 | China | GIIa |
| MT787025.1 | CH/SX/2016 | 2016 | China | GIIa |
| MH061340.1 | CH/SCZY103/2017 | 2017 | China | GIIa |
| MK644602.1 | L6-HB2017 | 2017 | China | GIIa |
| MK606369.1 | CH-HB2-2018 | 2018 | China | GIIa |
| MT090146.1 | CH/SXWS/2018 | 2018 | China | GIIa |
| MK644605.1 | T10-HB2018 | 2018 | China | GIIa |
| MT263014.1 | SC-YB73 | 2019 | China | GIIa |
| OM914738 | CH/HLJBQL/2022 | 2022 | China | GIIa |
| KF267450.1 | 13-019349 | 2013 | USA | GIIb |
| KF468754.1 | IA2 | 2013 | USA | GIIb |
| KF650370.1 | ISU13-19338E-IN-homogenate | 2013 | USA | GIIb |
| KJ662670.1 | KNU-1305 | 2013 | South Korea | GIIb |
| KF468752.1 | MN | 2013 | USA | GIIb |
| KJ778616.1 | NPL-PEDv/2013/P10 | 2013 | USA | GIIb |
| KR078300.1 | PC177 | 2013 | USA | GIIb |
| KM392231.1 | TC PC182-P2 | 2013 | USA | GIIb |
| KF452323.1 | USA/Indiana/17846/2013 | 2013 | USA | GIIb |
| KF804028.1 | USA/Iowa/18984/2013 | 2013 | USA | GIIb |
| KJ184549.1 | USA/KS/2013 | 2013 | USA | GIIb |
| KJ645658.1 | USA/Minnesota62/2013 | 2013 | USA | GIIb |
| KJ645640.1 | USA/Oklahoma32/2013 | 2013 | USA | GIIb |
| KJ645697.1 | USA/Texas128/2013 | 2013 | USA | GIIb |
| KJ645700.1 | MEX/124/2014 | 2014 | Mexico | GIIb |
| KR011756.1 | FR/001/2014 | 2014 | France | GIIc |
| LM645057.1 | L00721/GER/2014 | 2014 | Germany | GIIc |
| KJ399978.1 | OH851 | 2014 | USA | GIIc |
| KU847996.1 | ZL29 | 2015 | China | GIIc |

**Supplementary Table 3.** The primer sequences.

| Type of Virus | Name of Primer | Sequence of 5’-3’ | Target gene | Fragment length |
| --- | --- | --- | --- | --- |
| PEDV | S1-F | TACCTCCTACTGTCAGGGAAATTGTCA | S | 749bp |
|  | S1-R | GTCTGTGATACCTTCAAGTGGTTTAGG |  |  |
|  | ORF3-F | ATGTTTCTTGGACTTTTTC | ORF3 | 675bp |
|  | ORF3-R | TCATTCACTAATTGTAGCATAC |  |  |
|  | q-F | GCACTTATTGGCAGGCTTTGT | ORF3 | 100bp |
|  | q-R | CCATTGAGAAAAGAAAGTGTCGTAG |  |  |
| PCV2 | ORF2-F | CGGATATTGTAGTCCTGGTCG | ORF2 | 481bp |
|  | ORF2-R | ACTGTCAAGGCTACCACAGTC |  |  |
| PDCoV | N-F | ATGGCTACTGGCTGCGTTAC | N | 383bp |
|  | N-R | GCGTTTCCTGGGCTGATT |  |  |
| TGEV | S2-F | GTGGTTTTGGTYRTAAATGC | S | 859bp |
|  | S2-R | CACTAACCAACGTGGARCTA |  |  |
| PRRSV | Nsp2-F | ATGTTGTGCTTCCTGGGGTTG | Nsp2 | 600-1k bp |
|  | Nsp2-R | CTTGACAGGGAGCTGCTTGA |  |  |
| PBoV | NS1-F | ACAGGCAGCCGATCACTCACTAT | NS1 | 680bp |
|  | NS1-R | CTCGTTCCTCCCATCAGACACTT |  |  |
| PRV | gD-F | GGTGGACCGGCTGCTGAACGA | gD | 455bp |
|  | gD-R | GCTGCTGGTAGAACGGCGTCA |  |  |
| PKV | 3D-F | TGGACGACCAGCTCTTCCTTAAACAC | 3D | 443bp |
|  | 3D-R | AGTGCAAGTGCAAGTCTGGGTTGCAGCCA |  |  |
| BVDV | 5’UTR-F | GGTAGCAACAGTGGTGAG | 5’UTR | 220bp |
|  | 5’UTR-R | GTAGCAATACAGTGGGCC |  |  |


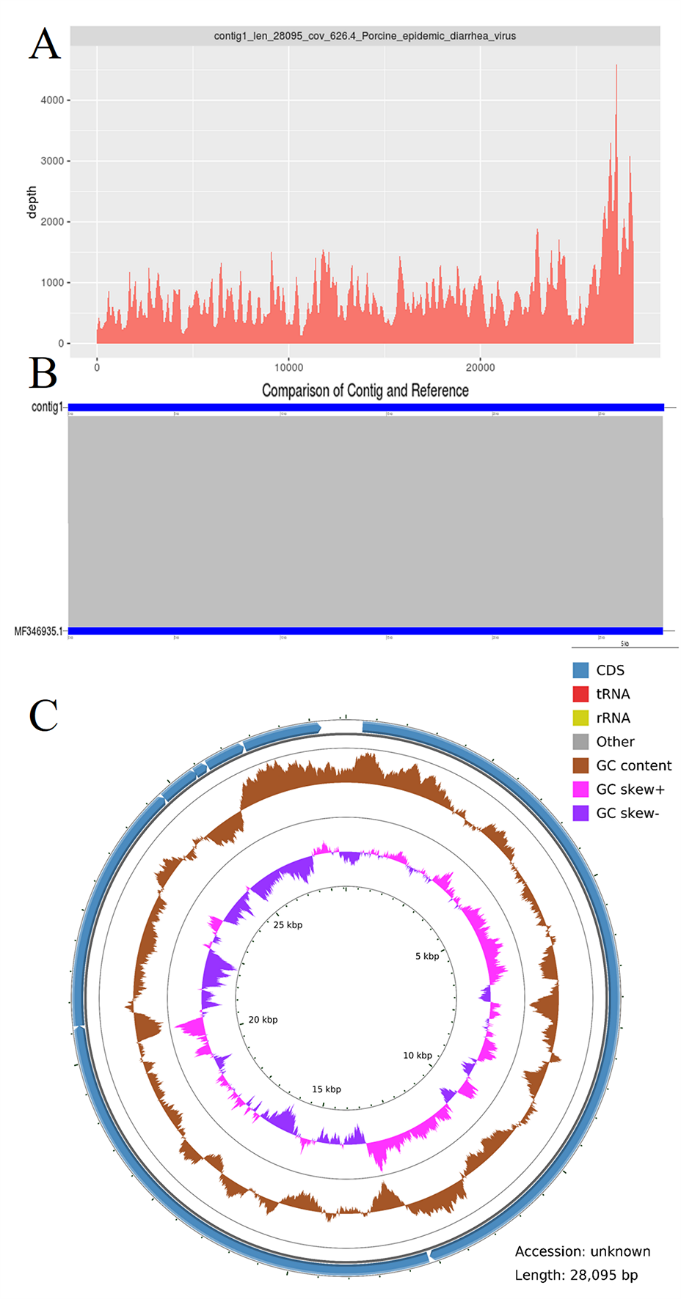


**Supplementary Fig. 1.** Sequencing results of CH/HLJBQL/2022. (A) Contigs depth statistical results are presented. (B) Best alignment results display. (C) The assembly result circle diagram exhibits. CDs: CDs fragment after assembled sequence annotation; GC content: display of GC content variation across assembled sequences (sliding windows of varying lengths were selected based on sequence length; contig length < 10000, sliding window length < 50; contig length < 100000, sliding window length 500); GC skew +/-: GC content offset, GC skew = (G - C) / (G + C), which measures the relative content of G and C, gives a positive value for GC skew if G > C and a negative value for G.


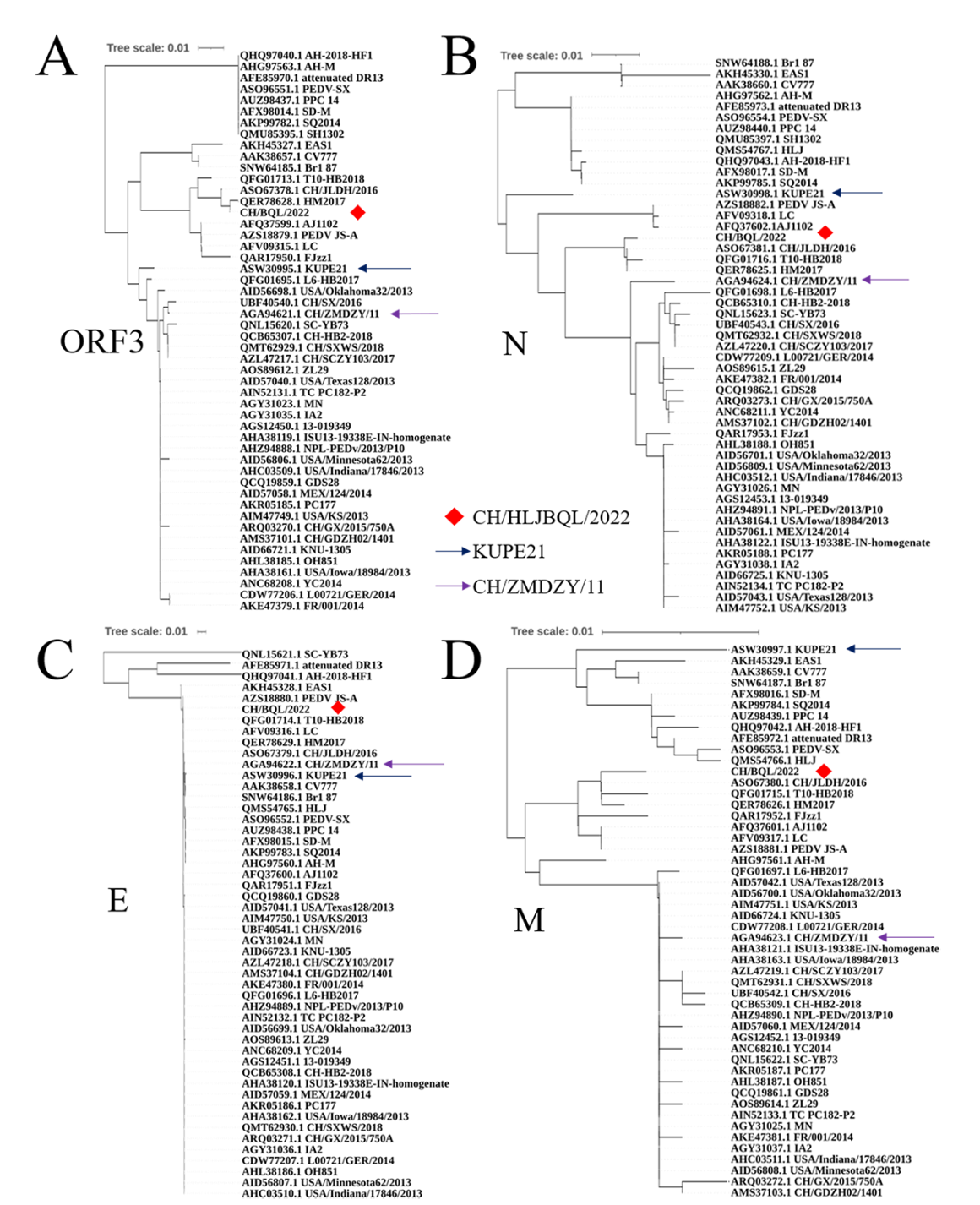


**Supplementary Fig. 2.** Evolutionary analysis of 51 PEDV strains. (A) Evolutionary analysis of ORF3 protein. CH/HLJBQL/2022 is marked in red, and arrows indicate KUPE21 (MF737355.1) and CH/ZMDZY/11 (KC196276.1) as early fusion strains. (B) Evolutionary analysis of N protein. (C) Evolutionary analysis of E protein. (D) Evolutionary analysis of M protein.


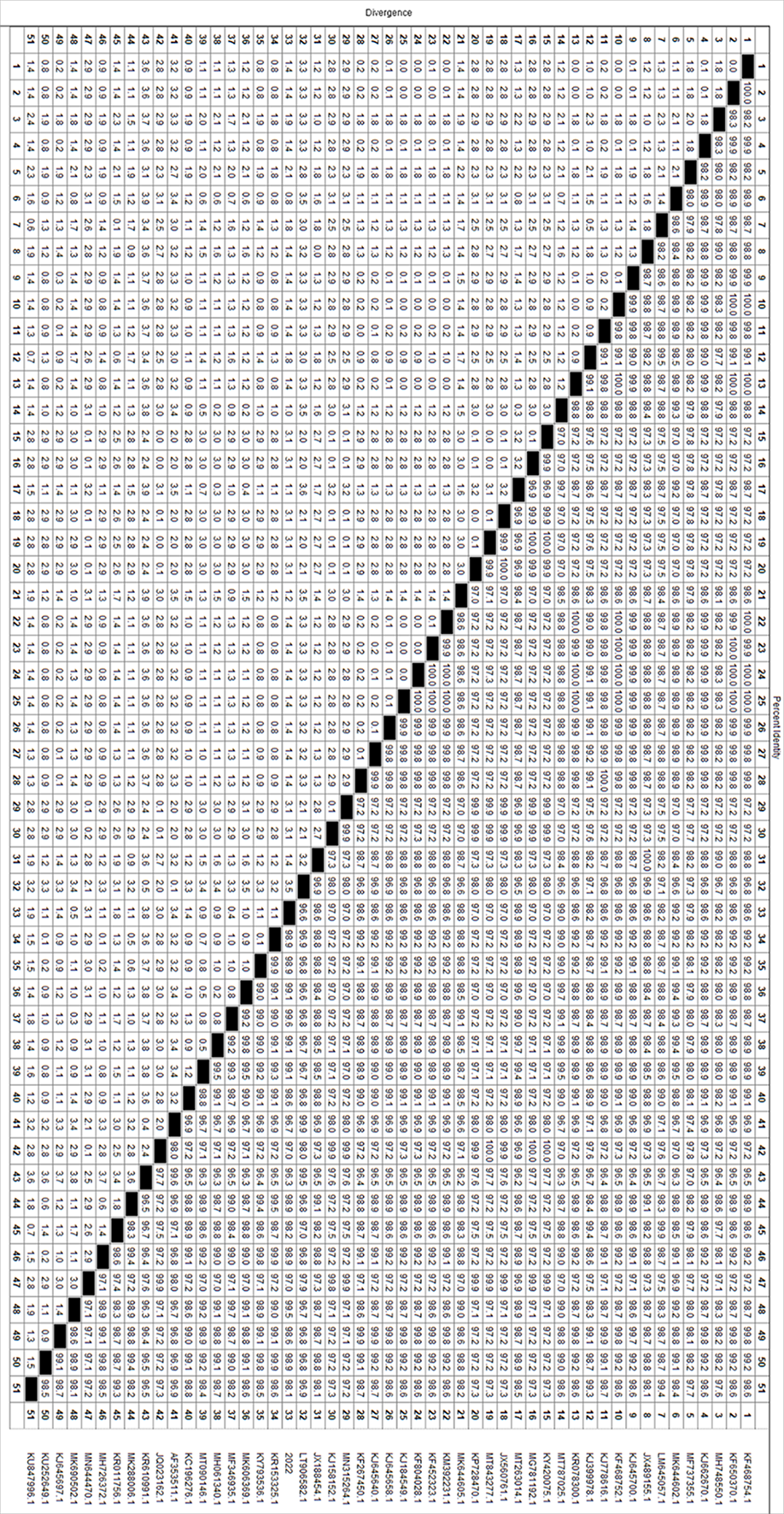


**Supplementary Fig. 3.** Sequence homology analysis of the whole genome of strain CH/HLJBQL/2022.


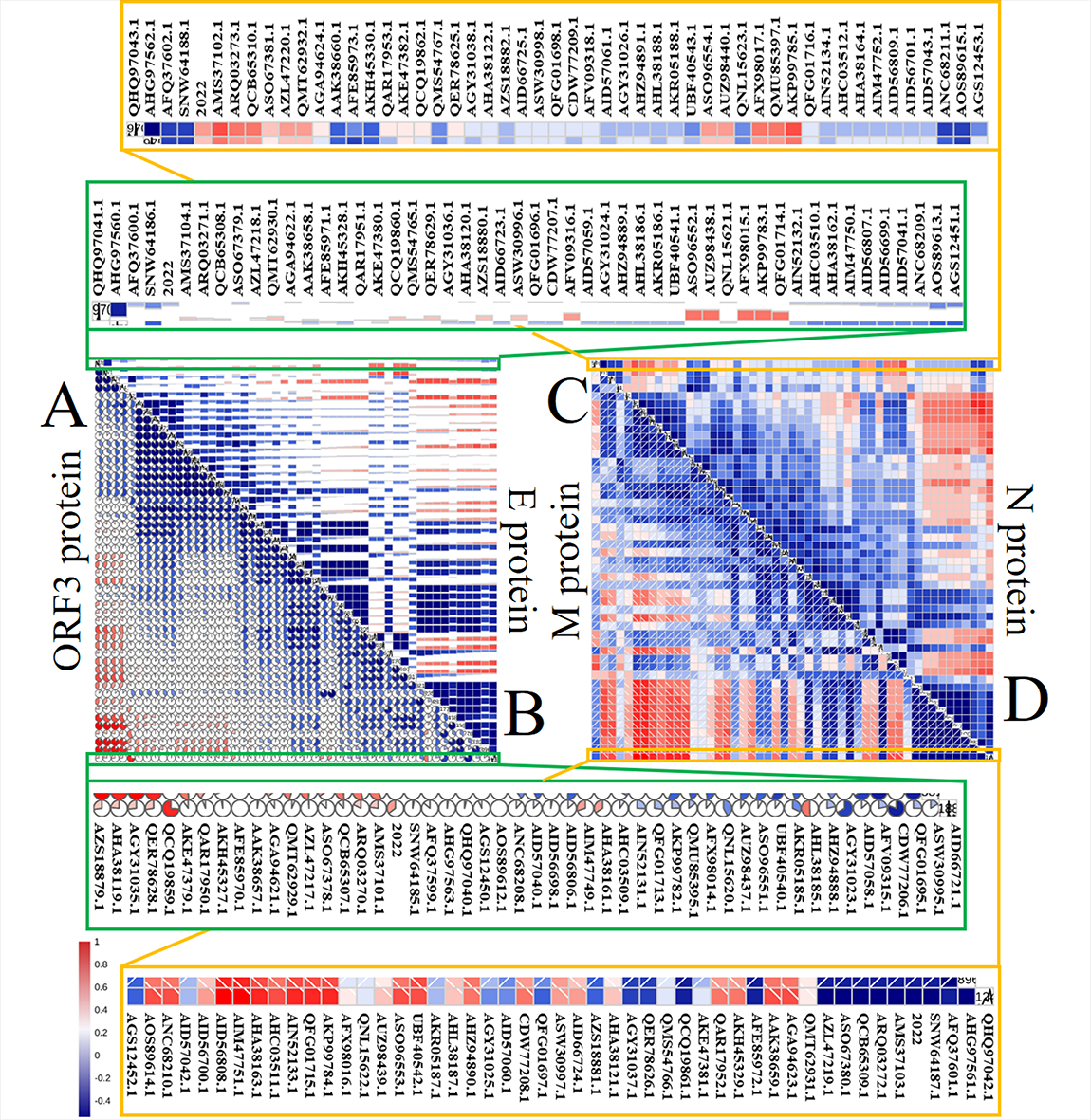


**Supplementary Fig. 4.** The homology of ORF3, E, M and N sequences of strain CH/HLJBQL/2022 was analyzed and displayed by heat map normalization. (A) Results of ORF3 gene sequence homology thermogram. (B) Results of E gene sequence homology thermogram. (C) Results of M gene sequence homology thermogram. (D) Results of N gene sequence homology thermogram.


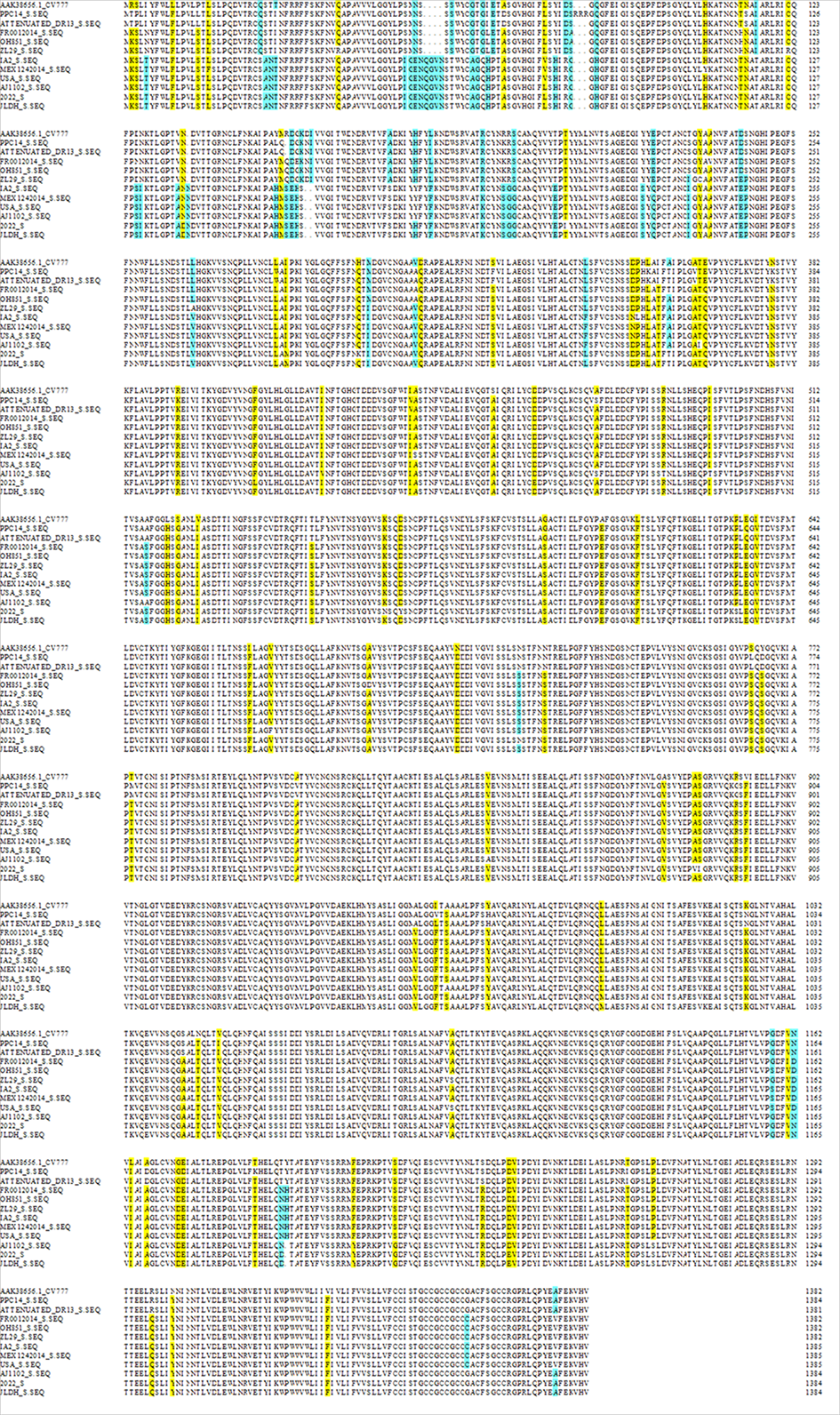


**Supplementary Fig. 5.** 11 representative strains and CH/HLJBQL/2022 strain S protein sequence alignment. CV777 (AF353511.1), PPC 14 (MG781192.1), attenuated DR13 (JQ023162.1), FR/001/2014 (KR011756.1), OH851 (KJ399978.1), ZL29 (KU847996.1), IA2 (KF468754.1), MEX/124/2014 (KJ645700.1), USA/Minnesota62/2013 (KJ645658.1), AJ1102 (JX188454.1) and CH/JLDH/2016 (MF346935.1) were aligned with reference.


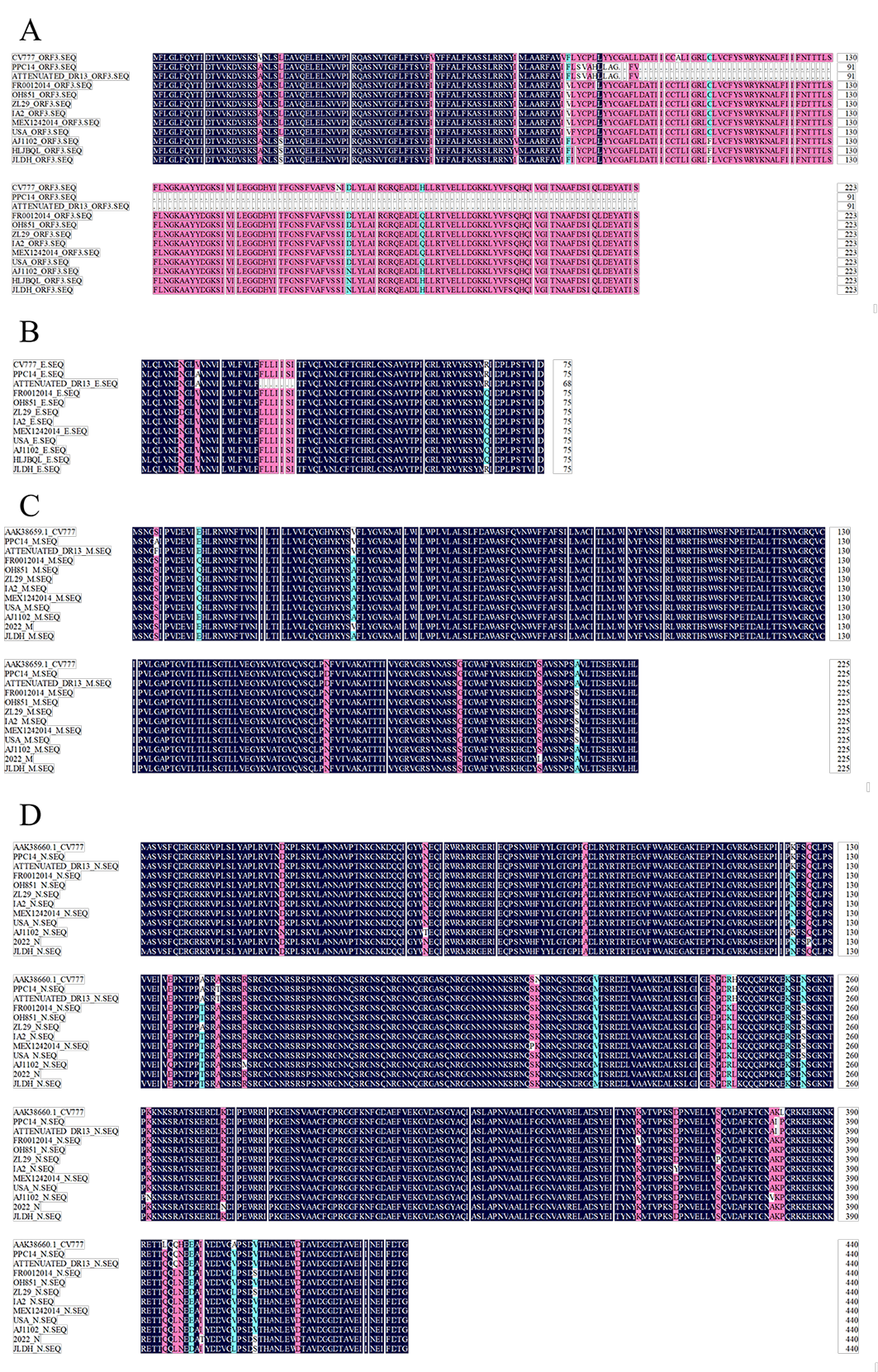


**Supplementary Fig. 6.** 11 representative strains and CH/HLJBQL/2022 strain other proteins sequence alignment. (A) Gene nucleotide contrast for ORF3. (B) Gene nucleotide contrast for E. (C) Gene nucleotide contrast for M. (D) Gene nucleotide contrast for N.


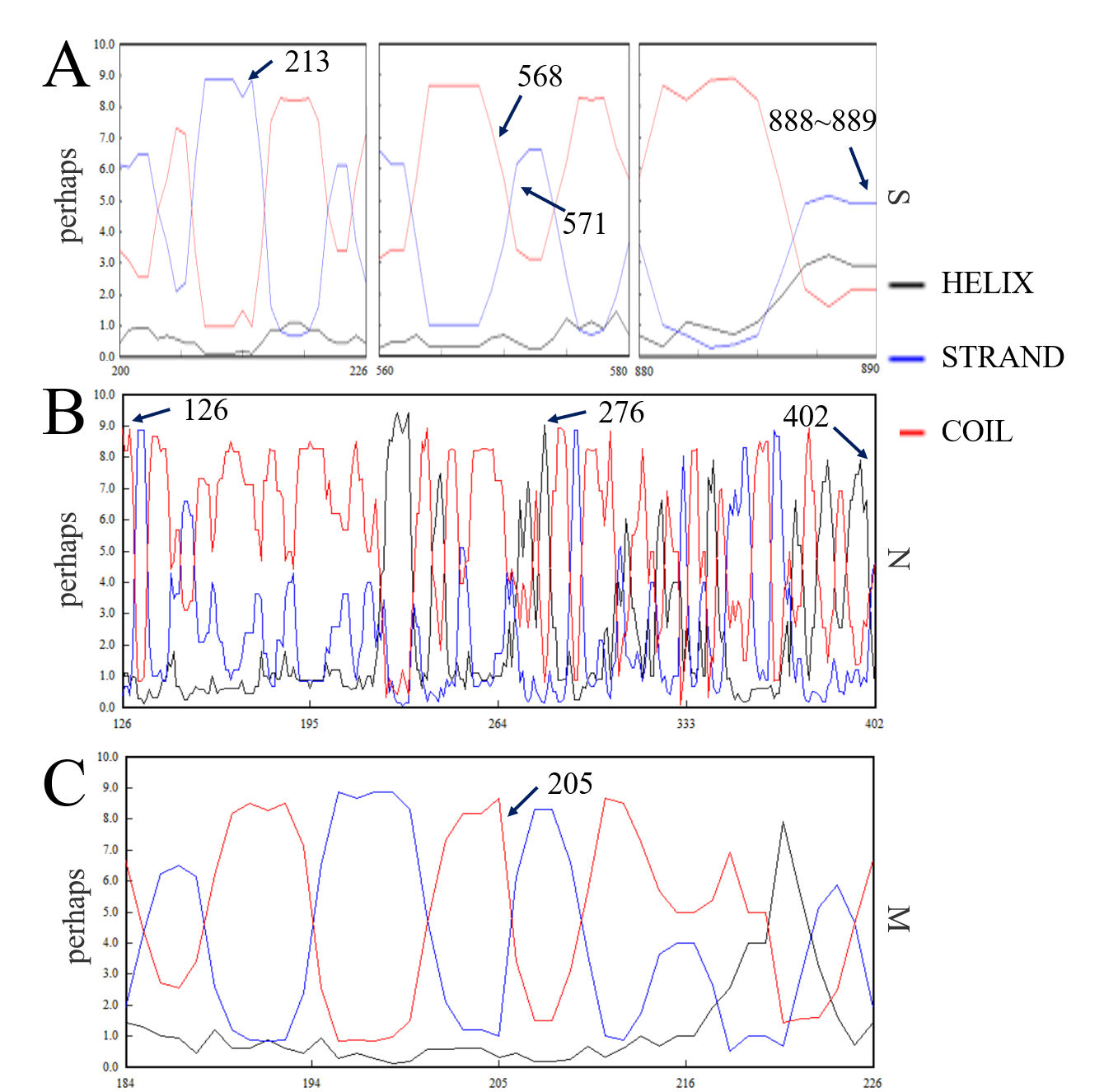


**Supplementary Fig. 7.** The secondary structure of S, N and M proteins was predicted. (A) The amino acid mutation sites were four predicted as strand and one as coil in the S protein. (B) The amino acid mutation sites were predicted to be helical in two helixes and coil in the N protein in one. (C) The site of the amino acid mutation was predicted to be coil in the M protein.


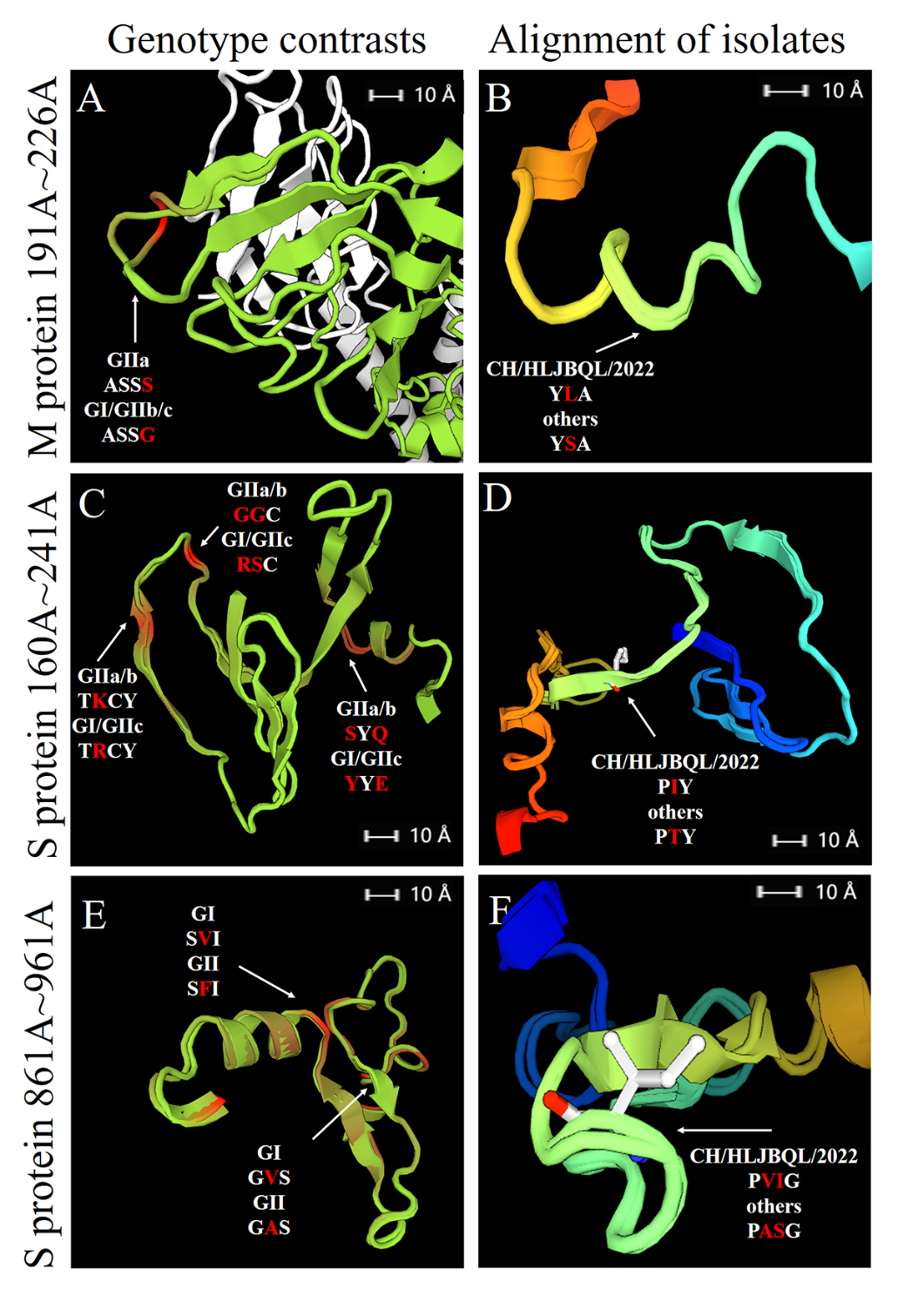


**Supplementary Fig. 8.** Tertiary structure modeling of mutation sites. (A) The ASSS - ASSG signature distinguishing GIIa from GI/GIIb/C is found in the M protein. This mutation, located at aa 191-226, produced a peculiar coil. (B) An S - L mutation (aa 207) in the M protein, found in the CH/HLJBQL/2022 strain, produced a peculiar coil. (C) The GGC - RSC, SYQ - YYE and TKCY - TRCY motifs GIIa/b and GI/GIIc distinctions are found in the S protein. These mutations located at aa 160-241 produced a peculiar coil with an extended last hallmark α Helix. (D) The S - L mutation (aa 213) found in the CH/HLJBQL/2022 strain that neutralizes an epitope in S1° of the S protein gives rise to a peculiar coil. (E) The distinction between SVI - SFI and GVS - GAS hallmarks GI and GII is found in the S protein. These mutations, located at aa 861 - 961, produced special coils and α Helix. (F) The VI - AS mutation (aa 888 - 889) in the S protein, found in the CH/HLJBQL/2022 strain, produced a special coil.
