# Supplementary material for: Based on the Results of PEDV Phylogenetic Analysis of the Most Recent Isolates in China, the Occurrence of Further Mutations in the Antigenic Site S1° and COE of the S Protein Which Is the Target Protein of the Vaccine
Source: Transbound Emerg Dis. 2023 Feb 22;2023:1227110. doi: 10.1155/2023/1227110 (PMC12016877; doi:10.1155/2023/1227110)
Supplement: Supplementary Materials — Supplementary Table 1. Recombinant plasmid sequences of PEDV ORF3. Supplementary Table 2. PEDV strains were used in this study. Supplementary Table 3. The primer sequences. Supplementary Figure 1 Sequencing results of CH/HLJBQL/2022. (A) Contig-depth statistical results are presented. (B) Best alignment results display. (C) The assembly result circle diagram exhibits. CDs: CDs fragment after assembled sequence annotation; GC content: the display of GC content variation across assembled sequences (sliding windows of varying lengths were selected based on sequence length; contig length < 10000, sliding window length < 50; contig length < 100000, sliding window length 500); GC skew±: GC content offset, GC skew = (G − C)/(G + C), which measures the relative content of G and C, gives a positive value for GC skew if G > C and a negative value for G. Supplementary Figure 2. Evolutionary analysis of 51 PEDV strains. (A) Evolutionary analysis of the ORF3 protein. CH/HLJBQL/2022 is marked in red, and arrows indicate KUPE21 (MF737355.1) and CH/ZMDZY/11 (KC196276.1) as early fusion strains. (B) Evolutionary analysis of the N protein. (C) Evolutionary analysis of E protein. (D) Evolutionary analysis of M protein. Supplementary Figure 3. Sequence homology analysis of the whole genome of strain CH/HLJBQL/2022. Supplementary Figure 4. The homology of ORF3, E, M, and N sequences of strain CH/HLJBQL/2022 was analyzed and displayed by heat map normalization. (A) Results of the ORF3 gene sequence homology thermogram. (B) Results of the E gene sequence homology thermogram. (C) Results of the M gene sequence homology thermogram. (D) Results of the N gene sequence homology thermogram. Supplementary Figure 5. 11 representative strains and CH/HLJBQL/2022 strain S protein sequence alignment. CV777 (AF353511.1), PPC 14 (MG781192.1), attenuated DR13 (JQ023162.1), FR/001/2014 (KR011756.1), OH851 (KJ399978.1), ZL29 (KU847996.1), IA2 (KF468754.1), MEX/124/2014 (KJ645700.1), USA/Minnesota62/2013 ( [file 1227110.f1.zip › Supplementary Table S1 (1).docx]

**Supplementary Table 1.** Recombinant plasmid sequences of PEDV ORF3.

| pMD19-ORF3: |
| --- |
| TCATTCACTAATTGTAGCATACTCGTCTAGTTGAATTGAGTCAAATGCAGCATTAGTAATGCCAACAATTTGATGTTGCGAAAAGACATAAAGCTTCTTGCCATCAAGAAGCTCAACAGTTCGCAACAGCTGTAGGTCGGCTTCTTGCCGCCCACGTATAGCTAGATACAAGTCAATGCTACTAACAAAAGCAACAAAAGAGTTGCCAAAAGTGATGTAATGGTCACCACCTTCTAAAATCACAATGGATTTGCCGTCATAATAAGCTGCTTTACCATTGAGGAAAGAAAGTGTCGTAGTATTAAAAATAATAAAGAGCGCATTTTTATAGCGCCAGGAGTAAAAGCAGACTAAACAAAGCCTGCCAATAAGTGTGCAACAAATAATAGTTGCATCTAAAAATGCACCACAATAATATAAAAGTGGGCAATAAAGAACAATGACAGCAAAACGCGCTGCCAACATAATATAATTGCGCCTCAAAGAAGACGCTTTAAACAGTGCAAAGAAGTAGATAAAAACACTGGTGAAAAGAAAACCCGTCACATTTGAAGCTTGTCTAATTGGAACTACATAGAGCTCCAACTCTTGGACAGCATCCAAAGACAAGTTAGCAGACTTTGAGACATCTTTGACAACTGTGTCAATCGTGTATTGAAAAAGTCCAAGAAACAT |
