# Supplementary material for: Based on the Results of PEDV Phylogenetic Analysis of the Most Recent Isolates in China, the Occurrence of Further Mutations in the Antigenic Site S1° and COE of the S Protein Which Is the Target Protein of the Vaccine
Source: Transbound Emerg Dis. 2023 Feb 22;2023:1227110. doi: 10.1155/2023/1227110 (PMC12016877; doi:10.1155/2023/1227110)
Supplement: Supplementary Materials — Supplementary Table 1. Recombinant plasmid sequences of PEDV ORF3. Supplementary Table 2. PEDV strains were used in this study. Supplementary Table 3. The primer sequences. Supplementary Figure 1 Sequencing results of CH/HLJBQL/2022. (A) Contig-depth statistical results are presented. (B) Best alignment results display. (C) The assembly result circle diagram exhibits. CDs: CDs fragment after assembled sequence annotation; GC content: the display of GC content variation across assembled sequences (sliding windows of varying lengths were selected based on sequence length; contig length < 10000, sliding window length < 50; contig length < 100000, sliding window length 500); GC skew±: GC content offset, GC skew = (G − C)/(G + C), which measures the relative content of G and C, gives a positive value for GC skew if G > C and a negative value for G. Supplementary Figure 2. Evolutionary analysis of 51 PEDV strains. (A) Evolutionary analysis of the ORF3 protein. CH/HLJBQL/2022 is marked in red, and arrows indicate KUPE21 (MF737355.1) and CH/ZMDZY/11 (KC196276.1) as early fusion strains. (B) Evolutionary analysis of the N protein. (C) Evolutionary analysis of E protein. (D) Evolutionary analysis of M protein. Supplementary Figure 3. Sequence homology analysis of the whole genome of strain CH/HLJBQL/2022. Supplementary Figure 4. The homology of ORF3, E, M, and N sequences of strain CH/HLJBQL/2022 was analyzed and displayed by heat map normalization. (A) Results of the ORF3 gene sequence homology thermogram. (B) Results of the E gene sequence homology thermogram. (C) Results of the M gene sequence homology thermogram. (D) Results of the N gene sequence homology thermogram. Supplementary Figure 5. 11 representative strains and CH/HLJBQL/2022 strain S protein sequence alignment. CV777 (AF353511.1), PPC 14 (MG781192.1), attenuated DR13 (JQ023162.1), FR/001/2014 (KR011756.1), OH851 (KJ399978.1), ZL29 (KU847996.1), IA2 (KF468754.1), MEX/124/2014 (KJ645700.1), USA/Minnesota62/2013 ( [file 1227110.f1.zip › Supplementary Table S2 (1).docx]

**Supplementary Table 2.** PEDV strains used in this study.

| Accession | Isolate | Collection Date | Geo Location | Genotype |
| --- | --- | --- | --- | --- |
| MF737355.1 | KUPE21 | 2001 | South Korea | - |
| KC196276.1 | CH/ZMDZY/11 | 2011 | China | - |
| AF353511.1 | CV777 | 1977 | Belgium | GI |
| LT906582.1 | Br1/87 | 1987 | United Kingdom | GI |
| JQ023162.1 | attenuated DR13 | 2009 | South Korea | GI |
| KJ158152.1 | AH-M | 2011 | China | GI |
| JX560761.1 | SD-M | 2012 | China | GI |
| MT843277.1 | SH1302 | 2013 | China | GI |
| KR610991.1 | EAS1 | 2014 | Thailand | GI |
| MG781192.1 | PPC 14 | 2014 | South Korea | GI |
| KP728470.1 | SQ2014 | 2014 | China | GI |
| MN644470.1 | HLJ | 2015 | China | GI |
| KY420075.1 | SX | 2015 | China | GI |
| MN315264.1 | AH-2018-HF1 | 2018 | China | GI |
| JX188454.1 | AJ1102 | 2011 | China | GIIa |
| MK288006.1 | FJzz1 | 2011 | China | GIIa |
| JX489155.1 | LC | 2011 | China | GIIa |
| MH726372.1 | GDS28 | 2012 | China | GIIa |
| MH748550.1 | JS-A | 2012 | China | GIIa |
| KR153325.1 | CH/GDZH02/1401 | 2014 | China | GIIa |
| KU252649.1 | YC2014 | 2014 | China | GIIa |
| KY793536.1 | CH/GX/2015/750A | 2015 | China | GIIa |
| MF346935.1 | CH/JLDH/2016 | 2016 | China | GIIa |
| MK690502.1 | HM2017 | 2016 | China | GIIa |
| MT787025.1 | CH/SX/2016 | 2016 | China | GIIa |
| MH061340.1 | CH/SCZY103/2017 | 2017 | China | GIIa |
| MK644602.1 | L6-HB2017 | 2017 | China | GIIa |
| MK606369.1 | CH-HB2-2018 | 2018 | China | GIIa |
| MT090146.1 | CH/SXWS/2018 | 2018 | China | GIIa |
| MK644605.1 | T10-HB2018 | 2018 | China | GIIa |
| MT263014.1 | SC-YB73 | 2019 | China | GIIa |
| OM914738 | CH/HLJBQL/2022 | 2022 | China | GIIa |
| KF267450.1 | 13-019349 | 2013 | USA | GIIb |
| KF468754.1 | IA2 | 2013 | USA | GIIb |
| KF650370.1 | ISU13-19338E-IN-homogenate | 2013 | USA | GIIb |
| KJ662670.1 | KNU-1305 | 2013 | South Korea | GIIb |
| KF468752.1 | MN | 2013 | USA | GIIb |
| KJ778616.1 | NPL-PEDv/2013/P10 | 2013 | USA | GIIb |
| KR078300.1 | PC177 | 2013 | USA | GIIb |
| KM392231.1 | TC PC182-P2 | 2013 | USA | GIIb |
| KF452323.1 | USA/Indiana/17846/2013 | 2013 | USA | GIIb |
| KF804028.1 | USA/Iowa/18984/2013 | 2013 | USA | GIIb |
| KJ184549.1 | USA/KS/2013 | 2013 | USA | GIIb |
| KJ645658.1 | USA/Minnesota62/2013 | 2013 | USA | GIIb |
| KJ645640.1 | USA/Oklahoma32/2013 | 2013 | USA | GIIb |
| KJ645697.1 | USA/Texas128/2013 | 2013 | USA | GIIb |
| KJ645700.1 | MEX/124/2014 | 2014 | Mexico | GIIb |
| KR011756.1 | FR/001/2014 | 2014 | France | GIIc |
| LM645057.1 | L00721/GER/2014 | 2014 | Germany | GIIc |
| KJ399978.1 | OH851 | 2014 | USA | GIIc |
| KU847996.1 | ZL29 | 2015 | China | GIIc |
