# Supplementary material for: Based on the Results of PEDV Phylogenetic Analysis of the Most Recent Isolates in China, the Occurrence of Further Mutations in the Antigenic Site S1° and COE of the S Protein Which Is the Target Protein of the Vaccine
Source: Transbound Emerg Dis. 2023 Feb 22;2023:1227110. doi: 10.1155/2023/1227110 (PMC12016877; doi:10.1155/2023/1227110)
Supplement: Supplementary Materials — Supplementary Table 1. Recombinant plasmid sequences of PEDV ORF3. Supplementary Table 2. PEDV strains were used in this study. Supplementary Table 3. The primer sequences. Supplementary Figure 1 Sequencing results of CH/HLJBQL/2022. (A) Contig-depth statistical results are presented. (B) Best alignment results display. (C) The assembly result circle diagram exhibits. CDs: CDs fragment after assembled sequence annotation; GC content: the display of GC content variation across assembled sequences (sliding windows of varying lengths were selected based on sequence length; contig length < 10000, sliding window length < 50; contig length < 100000, sliding window length 500); GC skew±: GC content offset, GC skew = (G − C)/(G + C), which measures the relative content of G and C, gives a positive value for GC skew if G > C and a negative value for G. Supplementary Figure 2. Evolutionary analysis of 51 PEDV strains. (A) Evolutionary analysis of the ORF3 protein. CH/HLJBQL/2022 is marked in red, and arrows indicate KUPE21 (MF737355.1) and CH/ZMDZY/11 (KC196276.1) as early fusion strains. (B) Evolutionary analysis of the N protein. (C) Evolutionary analysis of E protein. (D) Evolutionary analysis of M protein. Supplementary Figure 3. Sequence homology analysis of the whole genome of strain CH/HLJBQL/2022. Supplementary Figure 4. The homology of ORF3, E, M, and N sequences of strain CH/HLJBQL/2022 was analyzed and displayed by heat map normalization. (A) Results of the ORF3 gene sequence homology thermogram. (B) Results of the E gene sequence homology thermogram. (C) Results of the M gene sequence homology thermogram. (D) Results of the N gene sequence homology thermogram. Supplementary Figure 5. 11 representative strains and CH/HLJBQL/2022 strain S protein sequence alignment. CV777 (AF353511.1), PPC 14 (MG781192.1), attenuated DR13 (JQ023162.1), FR/001/2014 (KR011756.1), OH851 (KJ399978.1), ZL29 (KU847996.1), IA2 (KF468754.1), MEX/124/2014 (KJ645700.1), USA/Minnesota62/2013 ( [file 1227110.f1.zip › Supplementary Table S3 (1).docx]

**Supplementary Table 3.** The primer sequences.

| Type of Virus | Name of Primer | Sequence of 5’-3’ | Target gene | Fragment length |
| --- | --- | --- | --- | --- |
| PEDV | S1-F | TACCTCCTACTGTCAGGGAAATTGTCA | S | 749bp |
|  | S1-R | GTCTGTGATACCTTCAAGTGGTTTAGG |  |  |
|  | ORF3-F | ATGTTTCTTGGACTTTTTC | ORF3 | 675bp |
|  | ORF3-R | TCATTCACTAATTGTAGCATAC |  |  |
|  | q-F | GCACTTATTGGCAGGCTTTGT | ORF3 | 100bp |
|  | q-R | CCATTGAGAAAAGAAAGTGTCGTAG |  |  |
| PCV2 | ORF2-F | CGGATATTGTAGTCCTGGTCG | ORF2 | 481bp |
|  | ORF2-R | ACTGTCAAGGCTACCACAGTC |  |  |
| PDCoV | N-F | ATGGCTACTGGCTGCGTTAC | N | 383bp |
|  | N-R | GCGTTTCCTGGGCTGATT |  |  |
| TGEV | S2-F | GTGGTTTTGGTYRTAAATGC | S | 859bp |
|  | S2-R | CACTAACCAACGTGGARCTA |  |  |
| PRRSV | Nsp2-F | ATGTTGTGCTTCCTGGGGTTG | Nsp2 | 600-1k bp |
|  | Nsp2-R | CTTGACAGGGAGCTGCTTGA |  |  |
| PBoV | NS1-F | ACAGGCAGCCGATCACTCACTAT | NS1 | 680bp |
|  | NS1-R | CTCGTTCCTCCCATCAGACACTT |  |  |
| PRV | gD-F | GGTGGACCGGCTGCTGAACGA | gD | 455bp |
|  | gD-R | GCTGCTGGTAGAACGGCGTCA |  |  |
| PKV | 3D-F | TGGACGACCAGCTCTTCCTTAAACAC | 3D | 443bp |
|  | 3D-R | AGTGCAAGTGCAAGTCTGGGTTGCAGCCA |  |  |
| BVDV | 5’UTR-F | GGTAGCAACAGTGGTGAG | 5’UTR | 220bp |
|  | 5’UTR-R | GTAGCAATACAGTGGGCC |  |  |
