# Supplementary material for: Based on the Results of PEDV Phylogenetic Analysis of the Most Recent Isolates in China, the Occurrence of Further Mutations in the Antigenic Site S1° and COE of the S Protein Which Is the Target Protein of the Vaccine
Source: Transbound Emerg Dis. 2023 Feb 22;2023:1227110. doi: 10.1155/2023/1227110 (PMC12016877; doi:10.1155/2023/1227110)
Supplement: Supplementary Materials — Supplementary Table 1. Recombinant plasmid sequences of PEDV ORF3. Supplementary Table 2. PEDV strains were used in this study. Supplementary Table 3. The primer sequences. Supplementary Figure 1 Sequencing results of CH/HLJBQL/2022. (A) Contig-depth statistical results are presented. (B) Best alignment results display. (C) The assembly result circle diagram exhibits. CDs: CDs fragment after assembled sequence annotation; GC content: the display of GC content variation across assembled sequences (sliding windows of varying lengths were selected based on sequence length; contig length < 10000, sliding window length < 50; contig length < 100000, sliding window length 500); GC skew±: GC content offset, GC skew = (G − C)/(G + C), which measures the relative content of G and C, gives a positive value for GC skew if G > C and a negative value for G. Supplementary Figure 2. Evolutionary analysis of 51 PEDV strains. (A) Evolutionary analysis of the ORF3 protein. CH/HLJBQL/2022 is marked in red, and arrows indicate KUPE21 (MF737355.1) and CH/ZMDZY/11 (KC196276.1) as early fusion strains. (B) Evolutionary analysis of the N protein. (C) Evolutionary analysis of E protein. (D) Evolutionary analysis of M protein. Supplementary Figure 3. Sequence homology analysis of the whole genome of strain CH/HLJBQL/2022. Supplementary Figure 4. The homology of ORF3, E, M, and N sequences of strain CH/HLJBQL/2022 was analyzed and displayed by heat map normalization. (A) Results of the ORF3 gene sequence homology thermogram. (B) Results of the E gene sequence homology thermogram. (C) Results of the M gene sequence homology thermogram. (D) Results of the N gene sequence homology thermogram. Supplementary Figure 5. 11 representative strains and CH/HLJBQL/2022 strain S protein sequence alignment. CV777 (AF353511.1), PPC 14 (MG781192.1), attenuated DR13 (JQ023162.1), FR/001/2014 (KR011756.1), OH851 (KJ399978.1), ZL29 (KU847996.1), IA2 (KF468754.1), MEX/124/2014 (KJ645700.1), USA/Minnesota62/2013 ( [file Supplementary Figure S4 (1).pdf › Supplementary Figure S4 (1).pdf]

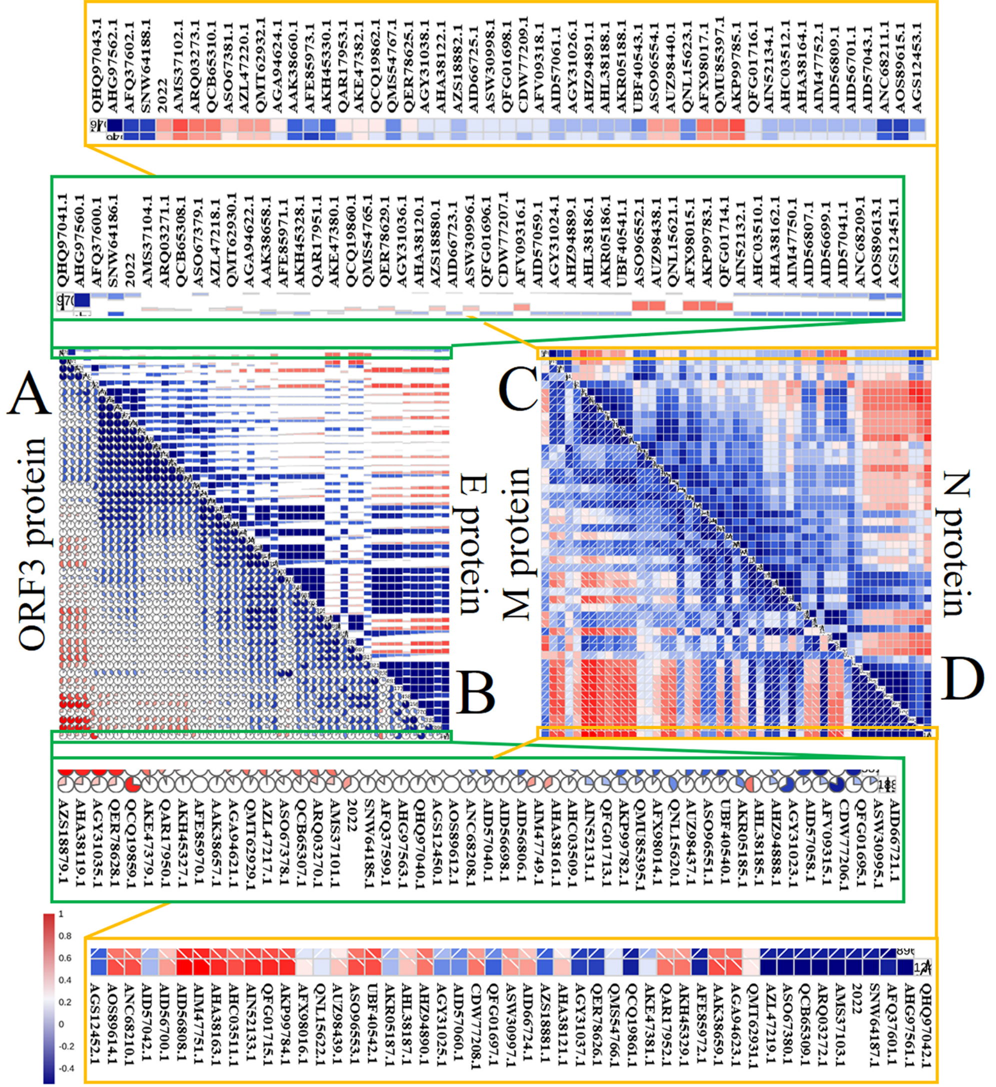
A
ORF3 protein
B
E protein
C
M protein
D
N protein
QHQ97043.1 AHG97562.1 AFQ37602.1 SNW64188.1 2022 AMS37102.1 ARQ03273.1 QCB65310.1 ASO67381.1 AZL47220.1 QMT62932.1 AGA94624.1 AAK38660.1 AFE85973.1 AKH45330.1 QAR17953.1 AKE47382.1 QCQ19862.1 QMS54767.1 QER78625.1 AGY31038.1 AHA38122.1 AZS18882.1 AID66725.1 ASW30998.1 QFG01698.1 CDW77209.1 AFV09318.1 AID57061.1 AGY31026.1 AHZ94891.1 AHL38188.1 AKR05188.1 UBF40543.1 ASO96554.1 AUZ98440.1 QNL15623.1 AFX98017.1 QMU85397.1 AKP99785.1 QFG01716.1 AIN52134.1 AHC03512.1 AHA38164.1 AIM47752.1 AID56809.1 AID56701.1 AID57043.1 ANC68211.1 AOS89615.1 AGS12453.1
QHQ97041.1 AHG97560.1 AFQ37600.1 SNW64186.1 2022 AMS37104.1 ARQ03271.1 QCB65308.1 ASO67379.1 AZL47218.1 QMT62930.1 AGA94622.1 AAK38658.1 AFE85971.1 AKH45328.1 QAR17951.1 AKE47380.1 QCQ19860.1 QMS54765.1 QER78629.1 AGY31036.1 AHA38120.1 AZS18880.1 AID66723.1 ASW30996.1 QFG01696.1 CDW77207.1 AFV09316.1 AID57059.1 AGY31024.1 AHZ94889.1 AHL38186.1 AKR05186.1 UBF40541.1 ASO96552.1 AUZ98438.1 QNL15621.1 AFX98015.1 AKP99783.1 QFG01714.1 AIN52132.1 AHC03510.1 AHA38162.1 AIM47750.1 AID56807.1 AID56699.1 AID57041.1 ANC68209.1 AOS89613.1 AGS12451.1
AZS18879.1 AHA38119.1 AGY31035.1 QER78628.1 QCQ19859.1 AKE47379.1 QAR17950.1 AKH45327.1 AFE85970.1 AAK38657.1 AGA94621.1 QMT62929.1 AZL47217.1 ASO67378.1 QCB65307.1 ARQ03270.1 AMS37101.1 2022 SNW64185.1 AFQ37599.1 AHG97563.1 QHQ97040.1 AGS12450.1 AOS89612.1 ANC68208.1 AID57040.1 AID56698.1 AID56806.1 AIM47749.1 AHA38161.1 AHC03509.1 AIN52131.1 QFG01713.1 AKP99782.1 QMU85395.1 AFX98014.1 QNL15620.1 AUZ98437.1 ASO96551.1 UBF40540.1 AKR05185.1 AHL38185.1 AHZ94888.1 AGY31023.1 AID57058.1 AFV09315.1 CDW77206.1 QFG01695.1 ASW30995.1 AID66721.1
1
0.8
0.6
0.4
0.2
0
-0.2
-0.4
AGS12452.1 AOS89614.1 ANC68210.1 AID57042.1 AID56700.1 AID56808.1 AIM47751.1 AHA38163.1 AHC03511.1 AIN52133.1 QFG01715.1 AKP99784.1 AFX98016.1 QNL15622.1 AUZ98439.1 ASO96553.1 UBF40542.1 AKR05187.1 AHL38187.1 AHZ94890.1 AGY31025.1 AID57060.1 CDW77208.1 QFG01697.1 ASW30997.1 AID66724.1 AZS18881.1 AHA38121.1 AGY31037.1 QER78626.1 QMS54766.1 QCQ19861.1 AKE47381.1 QAR17952.1 AKH45329.1 AFE85972.1 AAK38659.1 AGA94623.1 QMT62931.1 AZL47219.1 ASO67380.1 QCB65309.1 ARQ03272.1 AMS37103.1 2022 SNW64187.1 AFQ37601.1 AHG97561.1 QHQ97042.1
